# Therapeutic potential of mangiferin in cancer: Unveiling regulatory pathways, mechanisms of action, and bioavailability enhancements – An updated review

**DOI:** 10.1002/fsn3.3869

**Published:** 2023-12-20

**Authors:** Humaira Iqbal, Muhammad Inam‐Ur‐Raheem, Seemal Munir, Roshina Rabail, Sadia Kafeel, Arashi Shahid, Amin Mousavi Khaneghah, Rana Muhammad Aadil

**Affiliations:** ^1^ National Institute of Food Science and Technology University of Agriculture Faisalabad Pakistan; ^2^ Department of Fruit and Vegetable Product Technology Prof. Wacław Dąbrowski Institute of Agricultural and Food Biotechnology – State Research Institute Warsaw Poland

**Keywords:** angiogenesis, anticancer, anti‐inflammatory, antiproliferative, apoptosis, mangiferin

## Abstract

Mangiferin (MGF) is a phenolic compound, which is a major source of MGF is the mango tree. MGF possesses some antioxidant, anti‐inflammatory, and cytoprotective properties, enabling it to play its role against various diseases such as diabetes, obesity, lung injuries, and cancer. The word “Cancer” depicts an uncontrolled and abnormal growth of cells. This review paper reveals MGF's therapeutic, curative and protective potential impact against lung, liver, ovarian, prostate, breast, stomach, and oral cancers. MGF is used in various types of research in the form of powder, liquid extract, intramuscular, intravenous, nanoparticles coated with gold, in the form of a solution, or in combination with other drugs to evaluate synergistic effects. Many studies showed that MGF is safe to use but has less bioavailability in the body and 0.111 mg/mL solubility in water. However, certain studies indicated that its bioavailability and retention time increased when taken in the form of nanoparticles and in combination with other drugs. MGF also increases the sensitivity of other drugs (i.e., cisplatin) resistant to tumors. MGF has different mechanisms of action for different cancers. It mainly targets enzymes, interleukins, tumor growth factors, signaling pathways, apoptotic proteins, and genes to inhibit the growth of tumors, volume, angiogenesis, cellular functionality, further progression, and movement to other areas of the body. Moreover, MGF increases apoptosis and body weight with no or fewer side effects on normal cells. MGF unveiled a novel gate toward the treatment of cancer. Further research and human trials are needed in this regard.

## Graphical abstract

This review paper reveals MGF's therapeutic, curative and protective potential against lung, liver, ovarian, prostate, breast, stomach, and oral cancers. MGF is used in various types of research in the form of powder, liquid extract, intramuscular, intravenous, and nanoparticles coated with gold, in the form of a solution, or in combination with other drugs to evaluate synergistic effects.

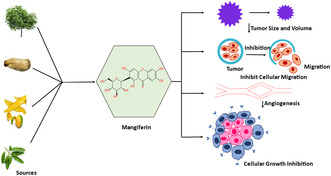

## 1 INTRODUCTION

The mangiferin (MGF) is classified as a phenolic compound and a C‐glycosylated xanthone (1,3,6,7‐tetrahydroxyxanthone‐C_2_‐β‐d glucoside), which is the branch of xanthone an organic compound. The first origin of MGF was *Anemarrhena asphodeloides* Bunge, which was greatly used in traditional Chinese medicine to treat neuropathy caused by diabetes (Jabeen et al., 2023; Lin et al., 2020). The major plant source of MGF is *Mangifera indica* (Family: *Anacardiaceae*), commonly named as mango tree; fruit shells, stem, greenery, bark, roots, and seed (Aboyewa et al., 2021; Liu et al., 2021; Rodriguez‐Gonzalez et al., 2021; Yadav et al., 2022) and also found in a few other medicinal herbs and families like *Salacia Chinensis* (*roots*) (Zeng et al., 2020), *Swertia chirata*, *Hypericum aucheri*, *Cyclopia intermedia* (Aboyewa et al., 2021; Rodriguez‐Gonzalez et al., 2021), *Anacardiaceae*, *Celastraceae*, *Gentianaceae families* (Zeng et al., 2020), *Zingiberaceae*, *Aphloiaceae Cnidii Rhizoma*, *Angelica Sinensis*, and *Levisticum officinale* (Grauzdytė et al., 2019) an *Aquilaria crassna* (agarwood) (Thitikornpong et al., 2019) and honeybush tea (Chen et al., 2022; Zhang & Wang, 2018).

The mango plant is grown throughout the world. At the same time, its crops are most common in tropical climates and subtropical climes (Lo Galbo et al., 2021), as well as in stifling areas of India, Africa, Asia, and Central America. MGF has grown in India's subcontinent for the last 4000 years and is abundantly used in Ayurvedic and traditional medical systems (Delgado‐Hernández et al., 2020). Among these are the Southern sections of the Italian Peninsula, such as Sicily, where mangoes have been grown for the past decade due to suitable pedoclimatic conditions (Lo Galbo et al., 2021). The major components of *C. intermedia* (a North American plant) extracts are xanthones (MGF) and flavanones (hesperetin and hesperidin), which are thought to be responsible for the majority of its pharmacological effects, including antidiabetic, anticancer, anti‐obesity, antioxidant, antimicrobial properties (Aboyewa et al., 2021; Wani et al., 2022), and radioprotective activities (Shang et al., 2021).

**FIGURE 1:**
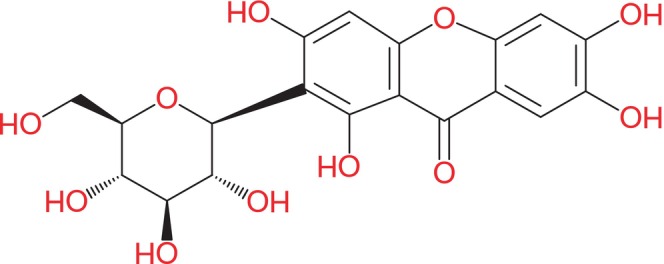
Structural formula of MGF (molecular formula: C_19_H_18_O_11_).

MGF was widely employed in ancient Chinese medications due to its pharmaceutical activities (Rodriguez‐Gonzalez et al., 2021; Xiao et al., 2021). The high content of macronutrients (carbohydrates and lipids), micronutrients (vitamins and minerals), and bioactive components (phytochemicals or polyphenols) in mango fruit gives it a high nutraceutical value. Several studies have been conducted in recent years employing the fruit's various components: peel, pulp, seed, and phytochemicals (Lo Galbo et al., 2021). Various studies show that MGF possesses some antioxidant actions, a role in treating obesity, the therapeutic potential for osteoarthritis (Aboyewa et al., 2021) protection of the gastrointestinal tract, an antidiabetic role, and in vitro repressing properties on type‐II 5α‐reductase, modulatory response in the immune system, that is, maybe suppressive or stimulative, hepatoprotective, and cardioprotective effects in various diseases in rodents (Khoobchandani et al., 2021; Liu et al., 2021; Mubashir et al., 2022; Xiao et al., 2021). It also shows photoprotective, analgesic, neuroprotective, anti‐Alzheimer (Grauzdytė et al., 2019), and anti‐allergic potential (Meng et al., 2023; Thitikornpong et al., 2019). The solubility of water for MGF is barely 0.111 mg/mL, which is much lower, limits its practical use, and consequently, no foodstuffs are in the marketplace. Therefore, it is essential to scrutinize a project and particular distribution system for MGF to augment its medicinal effectiveness (Xiao et al., 2021). Figure 1 explains the structural presentation of MGF.

The word “cancer” portrays cells abandoned and anomalous growth, which can also disturb other organs. The dissemination of annoying growth of cancerous cells is known as metastasis, which may contribute to fatality. According to a study, cancer has the highest mortality rate, that is, responsible for 7 million deaths yearly (Plonski et al., 2021; Saeed et al., 2022; Sudduth et al., 2023). The most common cancers are prostate, skin melanoma, colon, breast, and uterine (Carminatti et al., 2023; Miller et al., 2019; Saeed et al., 2022). Cancer can be caused due to some internal and external factors. The internal agent is genetics, and the external one includes biological, chemical, and physical carcinogens, that is, bacteria or viruses, tobacco, alcohol, arsenic, ionizing, and ultraviolet radiations. Older, inactive people with poor eating habits are more vulnerable to cancer than younger ones. Factors that can play a vital role in cancer prevention are the least exposure to sunlight, proper vaccination of viral infections, avoiding air pollution, eating fresh vegetables and fruits, and doing some physical activity daily (Cao et al., 2022; Leopold et al., 2022; Mitsogiannis et al., 2022). The risk of cancer can be reduced through timely diagnosis; early cancer treatment can increase the chances of survival (Ferlay et al., 2021) and appropriate patient care. It is supposed that every 30 days delay in surgery of cancer patients can increase the death rate to 6%–8% (Malagón et al., 2022). A study on outdated and corresponding medicine, 80% of people in under developed countries use plant‐derived medications for health care (Rodriguez‐Gonzalez et al., 2021). Traditional Chinese medicine can replace chemotherapy for better results in treating cancer (Sakthivel et al., 2023; Xiang et al., 2019; Zhang, 2023) and reduce side effects observed in radiotherapy (Nabil et al., 2018). The number of phytochemical compounds filed to the Food and Drug Administration is significant in the oncological field. Several pieces of research demonstrated their effective antioncogenic, cytoprotective, non‐progressive, and anti‐invasive roles against various types of cancer cells by deficiently defined processes (El‐Dawy et al., 2022; Elsayed et al., 2022; Rodriguez‐Gonzalez et al., 2021; Tauseef ur et al., 2023). As an adjuvant to first‐line medical therapy, nutrition‐based strategies for preventing certain malignancies provide a significant advantage over presently utilized medical therapies (Saeed et al., 2022). In recent years, researchers have been particularly interested in the anticancer potential of natural chemicals obtained from fruits, plants, or chemically altered phytochemicals for use as fresh resources for developing novel cancer therapies (Adoho et al., 2022; Dai et al., 2023; Ijaz et al., 2023; Krishnaveni et al., 2023; Lo Galbo et al., 2021; Samota et al., 2023; Swantara et al., 2022). Carcinogenesis is a multi‐factorial process involving several signaling pathways, and multitargeted phytochemicals are a prospective therapeutic area in cancer. A high antioxidant capacity and immunomodulatory phytochemicals are receiving significant scientific and clinical attention in developing innovative therapeutics for resilient drug malignancies such as castration‐resistant prostate cancer (Drobner et al., 2023; Khoobchandani et al., 2021). Chemoprevention is a new treatment that involves suppressing malignant development using natural herbal‐based substances (Liu et al., 2021).

Recently, MGF was isolated from the pulp of mango and co‐administrated with the chemotherapeutic drug doxorubicin (DOX) to exhibit anticancer action against (Lo Galbo et al., 2021) several cancers, including the breast cells “Michigan Cancer Foundation‐7” cells (MCF‐7) (Aboyewa et al., 2021), “M.D. Anderson‐Metastatic Breast 231” (MDA‐MB231 cell line), lung “Adenocarcinomic human alveolar basal epithelial cells‐549 cells” (A‐549 cells), cervical malignancies (Lo Galbo et al., 2021; Xiao et al., 2021), leukemia (U‐937 human Myeloid cells) (Aboyewa et al., 2021), “T Lymphoblast cell line” (MOLT‐4 cells) according to recent studies (Lo Galbo et al., 2021; Xiao et al., 2021), and also in multiple myeloma, gastric cancer, prostate cancer, ovarian adenocarcinoma, and colon cancer (Prasad et al., 2023; Shang et al., 2021). This impact stimulated proapoptotic proteins, cell cycle seizure, and the downregulation of reactive oxygen species (ROS) (Lo Galbo et al., 2021). MGF has been shown to suppress cancer cell growth by inducing apoptosis. Also, it has been shown to influence apoptotic pathways by numerous targets (Aboyewa et al., 2021), reduce the deoxyribose nucleic acid DNA damage, and decrease the formation of the tumor as well as its further migration (Yu et al., 2019); its antitumor mode of action concerning neovascularization and tumor cell motility has yet to be thoroughly understood (Delgado‐Hernández et al., 2020). MGF protects the mice against infection by increasing the frequency of delayed‐type hypersensitivity reactions and the titer of humoral antibodies (Pourzafar et al., 2023; Shang et al., 2021).

## 2 OBJECTIVES AND COLLECTION OF DATA

This review article aims to investigate accessible scientific and evidence‐based research about MGF (a phenolic compound mainly found in leaves, kernel, seed, and stems of mango) therapeutic potential against various cancers in its different forms, like powder, nanoparticles, injectables, and aqueous extracts, and to evaluate their findings for the better treatment of cancers and inhibit the further progression of tumors. Its efficacy for weight gain in different experimental units, prevention of tumor formation, minimizing cancer motility, and its use with other chemotherapeutic drugs to increase their efficiency, bioavailability, and retention time in the body are also discussed in this review. The main purpose of the review is to explore the MGF's mechanism of action in different pathways of certain cancers and to reveal the most appropriate form of MGF with its best‐obtained results. The main objective of this review is to assess the beneficial and medicinal effects of MGF against cancer. The second is to obtain lower side effects on healthy cells and greater cytotoxic effects on tumor cells. The other objective is to evaluate appropriate methods to increase its bioavailability and solubility. The last objective is to unveil the pharmacological value of the bioactive component of mango waste in therapeutics. Moreover, current available scientific‐based literature on the harmless and operative application of MGF for cancer therapy against different human cancer cell lines was collected and thoroughly studied for this review article. For data collection, powerful keywords like “mangiferin” OR “cancers” AND “anticancer agent” OR “mangiferin and cancer” have been searched at highly advanced search engines such as Google Scholar, Wiley, Scopus, and Science Direct. All possible research from 2017 to 2022 on cancer and MGF has been collected. However, the data obtained for “Improving Bioavailability of MGF” have some old literature before 2018. This review explains how MGF can be used to treat different cancers with fewer side effects, acceptable doses, and best outcomes, and this collected and confined description has yet to be found in past accessed reviews.

## 3 IMPROVING THE BIOAVAILABILITY OF MGF

MGF has low solubility as well as less oral bioavailability. These factors limit its use in therapy. Some experiments aimed to improve its bioavailability. In research, nanoprecipitation and solvent evaporation were used to design soft self‐assembled nanoparticles encapsulated with phospholipid complexes (MPLC‐SNPs) to enhance the antioxidant and biopharmaceutical potential of MGF. The solvent evaporation method was used to prepare the mangiferin‐phospholipon 90H complex (MPLC), and a central composite design was used for optimization. The nanoprecipitation method was used for the conversion of MPLC to MPLC‐SNPs. In vivo, antioxidant studies and liver function tests showed that MPLC‐SNPs notably conserved the antioxidant marker and CCl4‐intoxicated liver marker enzymes compared to MGN‐SNPs. The oral bioavailability of MPLC‐SNPs was significantly increased tenfold by increasing the key pharmacokinetic parameters, including the area under the curve maximum serum concentration (*C*_max_) and time for reaching up to maximum concentration (*T*_max_). Consequently, MPLC‐SNPs can be employed as a nanovesicle delivery system to improve MGF's antioxidant and biopharmaceutical potential (Telange et al., 2021).

Additionally, another examination showed that the MGF‐loaded nanostructured lipid carriers (MGF‐NLC) method's physicochemical properties are characterized by particle size, polydispersity index, zeta potential, trapping efficiency, drug loading, morphological property, and crystalline nation. In vitro characteristics have been investigated with drug release from NLC gadgets, bodily stability, and corneal permeation via excised rabbit cornea. Moreover, in vivo, ocular tolerability is assessed through a modified Draize check and histological microscopy. Finally, the results showed a 5.69‐fold increase in ophthalmic bioavailability compared to the MGF solution (MGN‐SOL) (Liu et al., 2012). The aim of the existing is to research the pharmacokinetic profiles of MGF and mangiferin monosodium salt (MG‐Na) in rat plasma by way of ultraperformance liquid chromatography–tandem mass spectrometry (UPLC‐MS/MS), which was then compared between the two companies. A suitable excessive sensitivity and selectivity UPLC‐MS/MS technique was used to evaluate plasma pharmacokinetics in MGF and MG‐Na using carbamazepine as an inner trend. The consequences showed that the area under the curve for MG‐NA was more than MGF alone, and its bioavailability was 70%. There were statistically substantial variations in the pharmacokinetic parameters between MGF and MG‐Na after a single oral administration at 100 mg/kg (Guo et al., 2019).

In another experiment, supercritical antisolvent techniques were used to enhance the bioavailability of MGF. In this method, N, N‐dimethylformamide was used to work as a solvent and carbon dioxide as an antisolvent to make microparticles of MGF. Results indicated that the bioavailability of MGF had been increased when utilized in microparticles than free MGF (Liu, Liu, et al., 2020; Nurhadi et al., 2022). Another study shows that when MGF is used with a complex of soy and phospholipid, it gives better bioavailability. This MGF complex was given to male albino rats in two doses, that is, 30 and 60 mg/kg BW, respectively. A soy phospholipid complex of MGF resulted in 9.75 times increased bioavailability than pure MGF and improved elimination half‐life (*t*_1/2_ el) (Bhattacharyya et al., 2014). Table 1 explains “increasing bioavailability by using a suitable dose” of MGF for its extensive use in treating various diseases. MGF has low solubility in GIT. The research was conducted to improve its bioavailability and absorption by co‐administrating three different potential absorption enhancers with MGF, that is, sodium deoxycholate (30 mg/kg), TPGS (50 mg/kg), and Carbopol 974P (100 mg/kg). The most favorable results were obtained from Carbopol 974P in 7 times increased bioavailability. Carbopol 974P has some mucoadhesive and drug permeation properties at the paracellular level. Only Carbopol 974P is described in Table 1 because it showed the best effects (Xiaoli Wang et al., 2013). Crude MGF was compared with MGF‐phospholipid complex to estimate their oral bioavailability. Results suggested that the complex has more bioavailability than crude MGF and more solubility and permeability (Ma et al., 2014). An increase in the bioavailability of MGF can be seen in Table 1.

**TABLE 1:** Increasing the bioavailability of MGF by using a suitable dose.

Animal	Dose (mg/kg BW)	Method	Increase in bioavailability (folds)	Citations
Male and female albino rats	60	Nanoprecipitation and solvent evaporation technique	10	Telange et al. (2021)
White rabbits	20	MGF‐loaded nanostructured lipid carriers' method	5.69	Liu et al. (2012)
Male Sprague–Dawley (SD) rats	100	Ultraperformance liquid chromatography–tandem mass spectrometry	5.7	Guo et al. (2019)
Female SD rats	100	Supercritical antisolvent techniques	2.07	Liu, Liu, et al. (2020)
Male albino rats	30, 60	Phospholipid complex technique	9.75	Bhattacharyya et al. (2014)
Rats	100	Co‐administrative technique	7	Xiaoli Wang et al. (2013)
Male SD rats	25	MGF‐phospholipid complex	2.3	Ma et al. (2014)

## 4 MECHANISM OF ACTION

**FIGURE 2:**
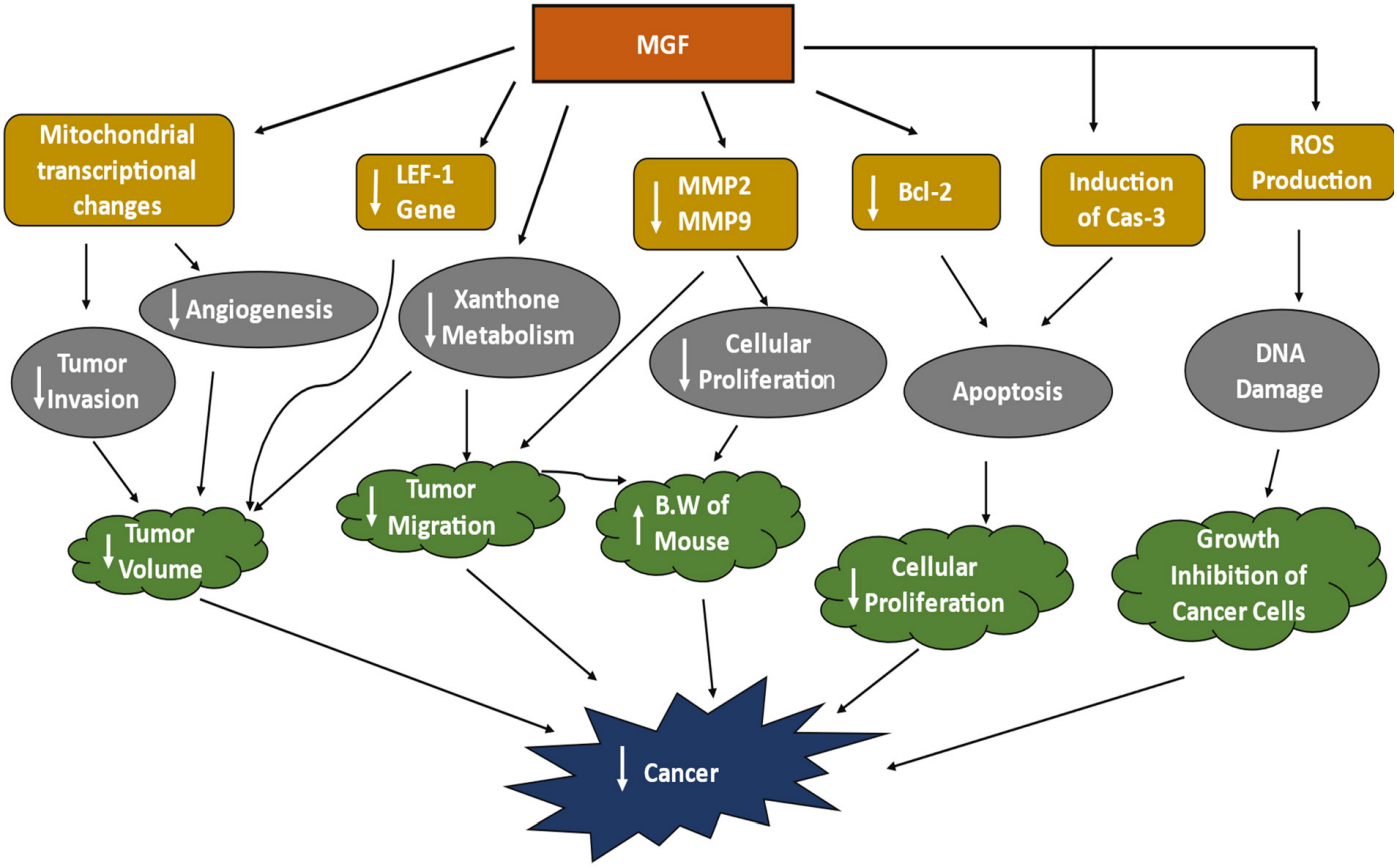
MGF mechanism against cancer.

**FIGURE 3:**
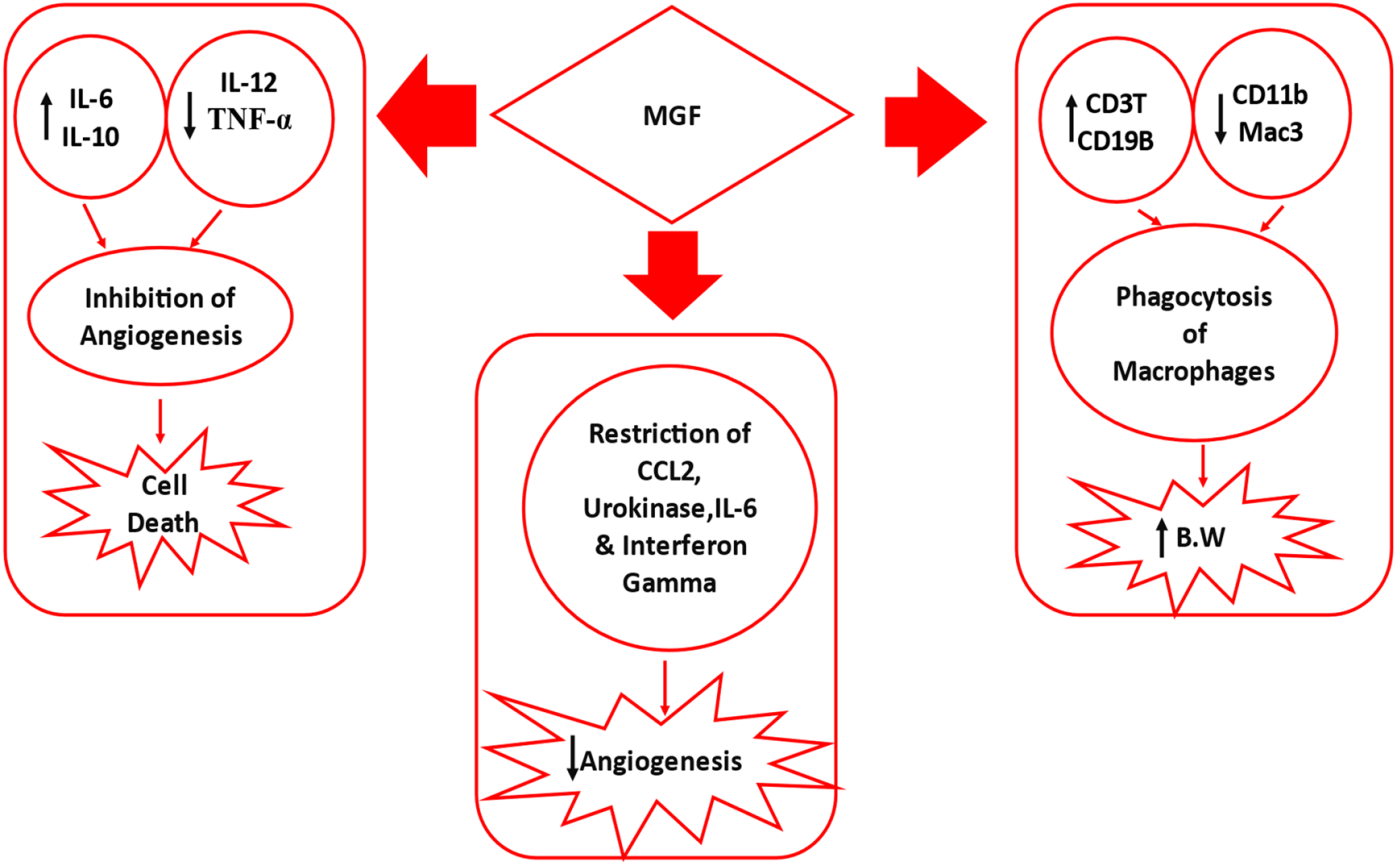
MGF modulation of the immune system.

**FIGURE 4:**
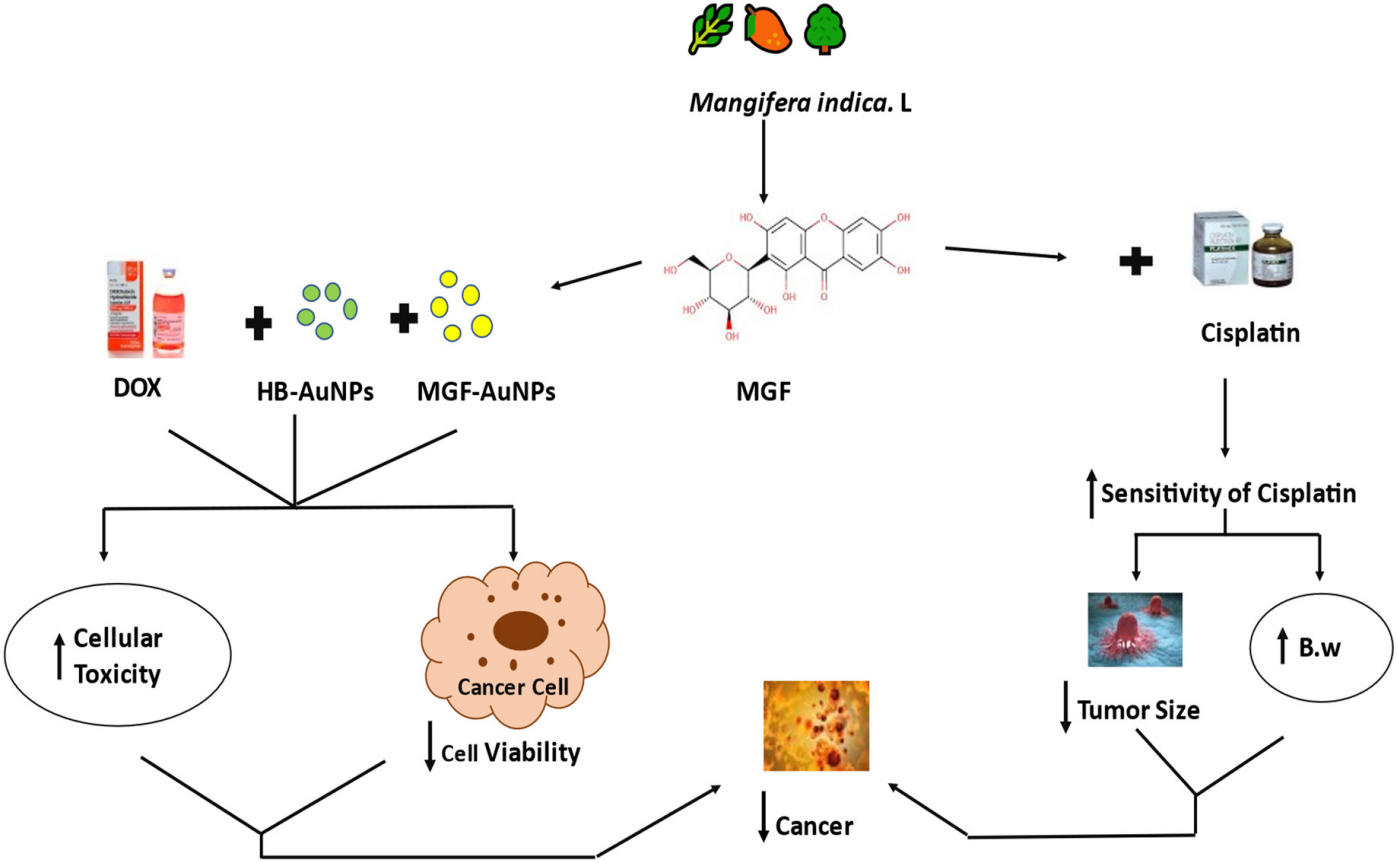
MGF's mechanism of action with other drugs.

The mechanistic action of MGF is supported by several evidence‐based researches. MGF has different mechanisms of action for different cancers. It depends upon the type of tumor and cancer and the dose of MGF. Here, we have concluded all the possible mechanisms of action in three ways. Moreover, reactions in each pathway are interconnected, as shown in Figures 2, 3, 4. In some studies, MGF causes changes in mitochondrial transcription, which may lead to decreased angiogenesis and tumor invasion. It also causes downregulation of Lymphoid enhancer‐binding factor‐1 (LEF‐1 gene) as well as decreases the xanthone (glucose) metabolism in tumor cells; all these actions result in a reduction of tumor volume (size) and decrease in tumor migration (movement to other cells) (Katti et al., 2018; Rodriguez‐Gonzalez et al., 2021; Tan et al., 2018). MGF causes downregulation of matrix metallopeptidase 2 (MMP‐2) and matrix metallopeptidase 9 (MMP‐9), which result in decreased cellular proliferation (progression) and tumor motility; these processes ultimately lead to an increase in body weight (Zeng et al., 2020). In one study, MGF causes downregulation of the B‐cell lymphoma (Bcl‐2) gene and triggers the induction of Cas‐3. These actions may play a role in apoptosis in cancerous cells. Furthermore, apoptosis causes a reduction in proliferation (Hu et al., 2022; Zhang & Wang, 2018). In another study, MGF increases the production of ROS, which may lead to DNA damage and ultimately inhibit the further growth of cancer cells (Yu et al., 2019). Ultimately, all the above results cause cancer suppression, as shown in Figure 2.

MGF brings modulations to the immune system to play its anticancer role. It increases the secretions of interleukin‐6 (IL‐6) and IL‐10. It decreases the IL‐12 and tumor necrosis factor‐alpha (TNF‐∝), which jointly inhibit the angiogenesis of cancer cells and result in cancer cell death (Hou et al., 2023; Khoobchandani et al., 2021; Sakthivel et al., 2023). In another study, it restricts c‐c Motif Chemo Ligand 2 (CCL2), urokinase (activator of plasminogen), IL‐6, and interferon‐gamma(INF‐γ), leading to a decrease in angiogenesis (Delgado‐Hernández et al., 2020). MGF causes an increase in a cluster of differentiation 3, which are co‐receptors of T‐cells (CD3T‐cells), and the cluster of differentiation 19 B, which are components of B cells (CD19B), as well as decreases CD11b monocytes, and Mac3‐macrophages (powerful macrophages) initiate phagocytosis of macrophages that lead to increase body weight and decrease in liver and spleen weights of the organism (Shang et al., 2021; Wen et al., 2022), as shown in Figure 3.

MGF has different mechanisms of action when used with other therapeutic drugs to treat cancer. In a study, gold‐coated nanoparticles of MGF (MGF‐AuNPs) and gold‐coated nanoparticles of Honeybush (HB‐AuNPs) were used with a chemotherapeutic drug named DOX, which collectively resulted in decreased cellular viability, that is, ability to work successively and increased cellular toxicity (Aboyewa et al., 2021). In other studies, MGF was used with Cisplatin, for example, an anticancer drug; it increased the sensitivity of cisplatin in the body and increased body weight while decreasing tumor size (He et al., 2019). Both these results have decreased cancer in the body, as shown in Figure 4.

## 5 ANTITUMOR EFFECTS OF MGF

Many types of research show that MGF has some antitumor effects against colorectal, oral, lung, ovarian, prostate, and hepatocellular tumors. Moreover, most studies are in vivo, but some are in vitro. Few studies contain both in vivo and in vitro examinations. In some studies, pure MFG was used, but it has less retention time and some side effects. MGF was also used with other compounds conjunctively to increase efficacy and decrease side effects. MGF also synergizes with other compounds to reduce tumor size and inhibit tumor growth (Siripongvutikorn et al., 2023).

### 5.1 In vivo studies

Several animals, like hamsters and male and female mice, were selected to study the antitumor effects of MGF. According to the 2018 Global Cancer Incidence, Mortality and Prevalence (GLOBOCAN) database, colorectal cancer (CRC) is the cause of 9% of cancer deaths and contributes to 10% of newly spotted cancer cases. CRC is both genders' most fatal and predominant tumor (Rodriguez‐Gonzalez et al., 2021). Moreover, CRC is the second most common cancer in females and the third in males (Bray et al., 2018; Ishibashi et al., 2021; Liu et al., 2022). MGF was obtained as a fluent extract of MGF from the mango tree's greeneries, woof, and trunk. The tumor was induced through N‐nitroso‐N‐methylurethane (NNMU) (a carcinogen) in cell line CT26. WT adenocarcinoma of the colon was then inoculated dermatologically on the right abaxial side of male Bagg and Albino Laboratory‐based Bred/colony (BALB/c) mouse of age 6–8 weeks and 18 to 22 g weight in all the experiments. Then, animals were divided into five groups, that is, ten experimental units/groups. Pure MGF was prepared in 0.05% carboxymethylcellulose (CMC) and Cis‐diamminedichloroplatinum (II) (CDDP; the generic name of cisplatin) in an antiseptic brine solution (0.9% sodium chloride) in different doses to the different groups, that is, 10, 50, and 100 mg/kg BW in a dose‐dependent manner to assess tumor growth by monitoring size, length, and volume of the tumor. The reference drug for MGF was CDDP, provided to one of the five groups. Reduction in tumor size starts at day 22 after inoculation of the tumor. Results indicate that the reduction in tumor size was near 75% in a dose‐dependent manner at 100 mg, and the overall reduction through reference drug (CDD) was 90%. 812 genes were downregulated at 100 mg/kg body weight MGF dose with minor transcriptional changes in mitochondrial metabolic strain, iron maintenance, fatty acid metabolism, peroxisome proliferator‐activated receptors, and regulators of lipid metabolism (PPARs), Glycoprotein 6 (GP6) signaling pathways and nuclear factor kappa B (NFkB) cause a decrease in movement (invasion) of the tumor and new blood vessels formation around tumor cells. MGF promotes decreased intra‐tumoral and pre‐tumoral blood supply levels (81% reduction at 200 μg/mL), and tumor volume and weight reduced by 84 and 73% at 200 μg/mL dose of MGF (Rodriguez‐Gonzalez et al., 2021).

Oral cancer is a malignant condition that includes head and neck carcinoma (HNC) (Liu et al., 2021); more than six lakh people have mouth cancer worldwide, and three lakhs die due to its severity. Risk factors for oral cancers are chewing betel nuts (Hung et al., 2020), overconsumption of tobacco, alcohol, and smoking. The tumor was induced through a carcinogen named 7,12‐dimethylbenz[a]anthracene (DMBA) in oral squamous cell carcinoma of hamsters aged 8–10 weeks and weighing 80–120 g at the time of the experiment. The total number of experimental units was 24(n), which (hamsters) were divided into four groups in a way that six animals/groups. Fourth group received alone‐MGF orally in a dose of 50 mg per kg body weight (of hamsters) without receiving DMBA, and third group was orally treated with 50 mg/kg BW MGF floating on corn oil in the very first week before the exposure to mutant (carcinogen) also continued till the 14th week of study. The second and third groups were treated with 0.5% DMBA from 2 to 10 weeks of the experiment. The study results show a significant increase in weight gain in the third group, weight reduction in the second group, and no change in body weight in the fourth group. It was also noted that in group 3, immature tumor formation occurred, and mature tumors were formed in group 2. In the plasma and oral mucosa of group 3 hamsters, the levels of antioxidant enzymes and nonenzymatic antioxidants were near to normal than in all other groups (Liu et al., 2021).

The prostate tumor is solid as it does not have cysts (Katti et al., 2018). Its incidence and death rates depend on biological location (Culp et al., 2020; Djiwa et al., 2022; Kimura & Egawa, 2018; Rawla, 2019). Previous research indicates that radiotherapy was used to treat prostate cancers. In this study, researchers used radioactive MGF‐198AuNPs instead of ionizing radioactive nanoparticles to check their antitumor effects on prostate cancer in men. Gold particles were produced through direct irradiation; one contained 1.55 mg of MGF. The findings revealed that these MGF‐198AuNPs cause an 85% reduction in tumor volume. The retention time of particles in the tumor also increased by more than 90% for a longer period with no leakage of nanoparticles through lymphatic ducts to non‐target tissues (organs), which is a most important feature. The results show that MGF‐198AuNPs contain greater tumoricidal properties than all other nanoparticles for prostate cancer (Katti et al., 2018). Liver cancer is the second largest leading cause of cancer deaths worldwide (after lung cancer). At the same time, hepatocellular carcinoma is the most dominant malignancy of the liver, responsible for 85% of all liver malignancies (Li et al., 2023). Previous research shows that hyperactivity of the Wingless/Integrated (Wnt) signaling pathway enhances tumors' constant growth and motility. MGF deactivates β‐catenin, which is a controller of Wnt signaling. In this study, Metastatic Hepatocellular Carcinoma Cell Line 97 (MHCC97L) of humans was used, which was tagged with luciferase (a reporter gene involved in oxidation). These cells were inoculated subcutaneously to the right side (between ribs and hips) of BALB/mouse. After the development of tumors, they were shifted to the right hemispheres of the livers of other mice of different species (*n* = 5). MGF dose was 50 mg/kg body weight for 2 days. Results indicated that MGF inhibits tumor growth (Tan et al., 2018). MGF was used to assess its effects on mammary carcinoma. For this purpose, rats were selected as experimental units. DMBA was administered via subcutaneous route using a 0.5 mL single dose in the mammary glands of rats to induce carcinogenesis. As a result of carcinogenesis, increased incidence and tumor volume were observed along with the decreased level of antioxidant enzymes, increased thiobarbituric acid reactive substance concentration in plasma, increased levels of NF‐κBp65, COX‐2, iNOS, that is, inflammatory markers, and increased expression of Bcl‐2 (antiapoptotic marker). When MGF was orally administered using 100 mg/kg BW/day, there was a significant reduction in the thiobarbituric acid reactive substance, and increased activity of antioxidant enzymes was seen. MGF inhibited NF‐κBp65 activation, thus conquering inflammation and proliferation, and it boosted proapoptotic proteins (Kari et al., 2022; Ullah et al., 2022; Xin Wang et al., 2021).

### 5.2 In vitro studies

Several cell lines were examined in in vitro experiments to check the antitumor effects of MGF. Surgical procedure is preferred over medicine to treat nonmetastatic colon cancer (Balasar et al., 2022; Coco & Leanza, 2022; Fei et al., 2018). The 95% pure MGF was used to assess its antitumor effects against CRC using cell line HT29 (ATCC HTB‐38) of Human Colon adenocarcinoma, and the culture medium was McCoy's 5. The concentration of HT29 cells was 35,000 cells/wells and treated with different applications of MGF like 1, 50, and 400 μM for cell feasibility assays. CT26 cells of mouse and HT29 human colon cells were treated with various MGF concentrations ranging from 10 to 400 μM. The results indicate no significant decrease or suppression of tumor invasion in HT29 cells. MGF overwhelms the vascular endothelial growth factor (VEGF) in the gene expression pathway, and it stimulates prometastatic motility in mouse cells and proangiogenic motility in human cells (Rodriguez‐Gonzalez et al., 2021).

The most dominant type of lung cancer is non‐small cell lung cancer (NSCLC), which is a major cause of cancer deaths, and the existence rate for up to 5 years is less than 20% (Jiang & Zheng, 2022; Lin et al., 2020). Additionally, black individuals with NSCLC are significantly less likely to undergo surgery, that is, 49 for stages I–II and 16 for stage III, compared to 55 and 22% for white ones (Miller et al., 2022). Its ancient treatments include surgery, chemotherapy, or radiation therapy. The cell lines used in the study were NCI‐H460 (HTB‐177), NCI‐H520 (HTB‐182), and A549(CCL‐185) for human large cell lung cancer, human squamous cell carcinoma, and non‐small cell carcinoma; 98% pure MGF was used in the study. The tumor was induced in cells through lipopolysaccharide (carcinogen). The in vitro results reveal that MGF inhibits the growth of tumor cells, inhibits expression of vimentin, decreases migration activity of NSCLC, decreases secretion of Interleukin 1 beta (IL‐1β), and reconciled protein levels of Period Circadian Regulator 1 (PER1) and NLR family pyrin domain containing 3 (NLRP3) in NSCLC (Lin et al., 2020). A comparative experiment was conducted to check the effectiveness of silver‐coated nanoparticles of MGF (AgNPs) over pure MGF against bone cancer (Osteosarcoma). AgNPs were synthesized, ranging from 25.30 to 40.79 nm in size. Different concentrations of pure MGF and AgNPs were utilized, that is, 10, 25, 50, and 100 μg/mL against Human MG‐63 (osteosarcoma cells) in rats. Results suggested that all AgNPs and pure MGF concentrations showed significant cytotoxic potential against cancerous cells. However, in the case of normal stem cells of mesenchyme, 50 μg/mL of pure MGF and 10 μg/mL of AgNPs were compared. Both concentrations are considered toxic to cancerous cells, but results indicated that these levels were safe for normal cells. Hence, AgNPs can be used to distribute MGF to cancerous cells better. AgNPs have more cytotoxic potential in bone cancer than pure MGF (Othman & Seka, 2019).

Ovarian cancer (OC) is the third most malignant and prevalent gynecological cancer and is ranked as the fifth cause of female cancer deaths (Liu, Xu, et al., 2020; Raoofi et al., 2022). However, it also has a high survival rate but relapses in about 70% of cases because of drug resistance. OVCAR8 cells of Human ovarian adenocarcinoma were used in this study, which was highly resistant to cadmium, cisplatin, and platinum chemotherapy (He et al., 2019). Cisplatin is the most efficient physiotherapeutic drug but has less sensitivity to the body (Buhari et al., 2023; Liu et al., 2018). The culture medium for tumor cells was Roswell Park Memorial Institute Medium‐1640 (RPMI‐1640). Cells were divided into three groups, one of them treated with 1 μg/mL of cisplatin, second with 25 μg/mL MGF, and third with both cisplatin (1 μg/mL) and MGF (25 μg/mL). The results showed that MGF increases the sensitivity (decrease resistance) of cisplatin to OVCAR8 cells, decreases tumor cell motility (migration), inhibits the tumor cell growth, and increases survival rate by downregulation (suppression) of yes‐associated protein (YAP) (He et al., 2019). The present treatment for liver cancer is liver transplant and surgery. The human lung fibroblasts (HLF) cell line was placed in a Dulbecco's modified Eagle (DMEM) culture. Results showed that MGF inhibits further growth and motility of tumors. The main target of MGF was Wnt signaling, and it downregulated LEF1. The overexpression of LEF1 works as an antagonist to MGF. The downregulated LEF 1 was not linked with β‐catenin but associated with Wilms' tumor 1 (WT 1) protein in a way that decreases the occupancy of WT1 at the promoter of LEF1 (Tan et al., 2018). Table 2 describes “anticancer action of MGF based on human cell line” studies to evaluate the therapeutic potential of MGF.

**TABLE 2:** Anticancer action of MGF based on human cell lines.

Cancer	Cell culture	Culture medium	MGF concentration	Results	Citations
Colorectal cancer	HT29 (ATCC HTB‐38) of human colon adenocarcinoma	McCoy's 5	10–400 μM	The motility of cancer cells was suppressed	Rodriguez‐Gonzalez et al. (2021)
Cervical, lung, and breast cancer	MCF‐7, A549, and Hela cell lines	RPMI 1640	25, 50, 75, 150, 300, 450, and 500 μM	Inhibited cellular proliferation, ↑retention time, and cytotoxicity	Xiao et al. (2021)
Triple‐negative breast cancer	BL21 (DE3) cells of *E. coli*	LB	90, 180, 360 μg	PKM2 activity ↓in a dose‐dependent manner	Rasul et al. (2021)
Colon, brain, and prostate cancer	Colon cell line (Caco‐2), glioblastoma cell line (U87) prostate cell line (PC‐3)	DMEM, RPMI 1640	15.62–1000 μg/mL	The efficiency of cells ↓from 40%–70% and toxicity was greater at higher concentrations	Aboyewa et al. (2021)
Lung cancer	Human NSCLC cell line A549 (CCL‐185), NCI‐H460 (HTB‐177) and squamous cell carcinoma cell line NCI‐H520 (HTB‐182)	RPMI 1640	3.12, 6.25, 12.5, 25, 50, 100, 200, 400, or 800 μg/mL	PER1 was less expressed in LPS‐stimulated NSCLC cells, MGF in combination increases PER1 expression	Lin et al. (2020)
Skin cancer	B16F10 (B16F10 cell line) melanoma and EA. hy 926 endothelial cells of the human umbilical vein	Medium (DMEM)	240 μM	Inhibition of cell migration, non‐cytotoxic effects, inhibition of endothelial growth, 50% inhibition in capillary growth, and 70% ↓ in tube formation	Delgado‐Hernández et al. (2020)
Pancreatic cancer	Mia‐PaCa2 and HTRET‐HPNE cells	DMEM	0–100 μM	Suppressed viability of cells, induced autophagy as well as cell death by apoptosis in Mia‐PaCa2 cells, ↑ROS levels, ↓motility of cancerous cells	Yu et al. (2019)
Bronchial cancer	Human bronchial epithelial cells BEAS‐2B, SV40‐adenovirus hybrid (Ad12SV40)	F‐12K	0.5 μg/mL of DMSO	Cell progression was reinstated, oxidative stress ↓ by using PAH & MGF, ↑ wound healing	Grauzdytė et al. (2019)
Colorectal, breast, and liver cancer	Human colorectal adenocarcinoma (HT29), hepatocellular carcinoma (HepG2), and human breast cancer cells (MDA‐MB‐231)	Complete medium	3 mg/mL	Inhibition of glucosidase activity, ↑scavenging activity of DPPH depending upon the dose, highest NO scavenging action (57%) at 60 μg/mL	Thitikornpong et al. (2019)
Ovarian cancer	Human ovarian adenocarcinoma OVCAR8 cells	RPMI‐1640	25 μg/mL	↑cell apoptosis, suppressed tumor cell movement, ↓levels of TEAD4 protein, improved sensitivity of tumor cells to cisplatin	He et al. (2019)
Thyroid cancer	TPC‐1 (human papillary thyroid cancer cell line)	DMEM‐F12	1, 2, 4, 6, 8, 10 μM	50% ↓ in cell numbers at 4 μM MGF, induction of cell death at 2 and 4 μM, ↓levels of antiapoptotic protein Bcl‐2	Zhang and Wang (2018)
Hepatocellular carcinoma	Human HCC cell line and MHCC97L HLF cell line	DMEM	120 μg/mL	↓cell proliferation, no alteration in transcription and translation of β‐catenin, suppressed *LEF1* expression by ↓binding WT1 to the *LEF1* promoter region	Tan et al. (2018)
Gastric cancer	SGC‐7901 and BCG‐823 gastric cell lines	N/A	N/A	↓growth, progression, and angiogenesis	Du et al. (2018)
Bone cancer	Human MG‐63 (osteosarcoma cells)	MEM/DMEM	10, 25, 50, and 100 μg/mL	↓cancerous cell growth	Othman and Seka (2019)

## 6 INHIBITORY ROLE AGAINST CANCEROUS CELLS

### 6.1 Inhibition of pyruvate kinase M2 (PKM2)

Previous research shows that PKM2 (an isoform of pyruvate kinase) acts as an ideal target for drugs used to treat cancers. It is an enzyme for glucose metabolism, and that glucose is used by cancer cells. It is upregulated in some cancers, disturbs the normal glycolytic activity of glucose, and helps in tumorigenesis, a natural suppressor of PKM2. It can be a novel cancer treatment (Choi et al., 2021; Rasul et al., 2021). Breast cancer is the second largest reported cancer in the United States after skin cancer and also the second leading cause of mortality after lung carcinoma but the first leading cause of death in Black women (Giaquinto, Miller, et al., 2022; Miller et al., 2021). It is more common in Black women than in any other. The incidence rate of breast cancer has increased (0.5% each year) in the last decade, but on the other side, its death rate has decreased (43% from 1989 to 2020) (Giaquinto, Sung, et al., 2022). The underlying causes of breast cancer are obesity, a sedentary lifestyle, and alcohol consumption (Islami et al., 2018). In this study, MGF extracts obtained from the bark, greens, and seed were found to be inhibitors of PKM2 through screening of the plant extract library. MDA‐MB231 human triple‐negative breast cancer (TNBC) cells were cultivated in (DMEM). MGF showed an inhibitory effect on the growth of TNBC. Different concentrations of 90, 180, and 360 μg/mL of MGF extracts were used in a concentration‐dependent manner to check the impact on PKM2 activity. Results indicated that PKM2 activity was lowest at 360 μg/mL. Ninety‐four compounds derived from MGF were tested against three binding sites of PKM2 to validate their suppressive action. This research unveiled the novel modulators of PKM2. Further confirmation is still required because many enzymes of glucose metabolism can be targeted, but this investigation focused on PKM2 (Rasul et al., 2021).

### 6.2 Inhibition of cellular proliferation and growth of cancerous cells

A study was designed to evaluate the effects of MGF against different cancers like cervical, breast, and lung cancers. Nanoparticles named magnetic microspheres loaded with MGF (MG‐MS) were compared with free MGF. MCF‐7, A549, and Hela cell lines were used in in vitro assays. Results indicated that MG‐MS inhibited cellular proliferation more than free MGF. Cytotoxicity of MGF increased due to the presence of MS, which may improve the solubility of MGF in water. The release of MGF from nanoparticles was prolonged, which is helpful in action. So, MS can be used as a carrier of MGF to treat certain tumors (Xiao et al., 2021).

A progress report shared by the American Cancer Society revealed that 22,530 new cases and 13,980 deaths of OC were reported in 2019. Epithelial carcinoma is the most prevalent and lethal form of OC. The main purpose of this study was to check the anticancer role of MGF against epithelial carcinoma; the powder was used, which was dissolved in dimethyl sulfoxide (DMSO at 6 mg/mL). One of the epithelial carcinoma cell walls was A2780, prepared in DMEM; the other was ES‐2, cultivated in McCoy's 5A (medium). Both in vitro and in vivo experiments were conducted to ensure the effects of MGF. In vitro, findings show that MGF decreases epithelial cell carcinoma's hiking (proliferation) depending on dose (more dose results in less proliferation). At the 300 μΜ doses of MGF, cancer cells' motility and migration were minimal. In an in vivo study, an ES‐2 cell line was inserted in mice with MGF doses, 60 mg/kg body weight (BW) and 20 mg/kg BW, respectively. Results revealed that a significant increase in the mouse's weight was observed, as well as a reduction in tumor growth and volume. In the enzyme‐linked Immunosorbent assay (ELISA) technique, it was clear that MGF caused the downregulation of MMP2 and MMP9 in cancerous cells to decrease their progression (Zeng et al., 2020). Pancreatic cancer is ranked number seven for cancer deaths and is difficult to treat due to drug resistance and the side effects of chemotherapy (Yu et al., 2019). After diagnosis, only 1/5 of patients are selected for resection to cure it, but cancer can recur in them within 2 years (AlMasri et al., 2022). Adjuvant therapy is used after the main treatment to destroy the leftover cancerous cells and is supposed to be the standard one to decrease the chances of recurrence (Conroy et al., 2018; Tempero et al., 2019). This research was conducted to check the anticancer effects of MGF against pancreatic cancer by using the Mia‐PaCa2 cell line and normal cell line HTRET‐HPNE to check how much MGF is deadly for them. Results indicated that MGF inhibited the growth of cancerous cells, and the inhibitory concentration of MGF for the cancerous cell was 10 μM, but it was 75 μM for normal cells. There were increased expressions of light chain 3 II (LC3 II), light chain 3 (LC3), and Beclin‐1 (Becn1), indicating self‐eating (autophagy) of Mia‐PaCa2 cells. MGF caused apoptosis (programmed cell death) due to the upregulation of proteins Bcl‐2 and Bax, which act as apoptosis biomarkers. Moreover, MGF increases the endogenous production of ROS at 20 μM concentration, which causes damage to the DNA of cancer cells. A significant reduction in mitochondrial‐mediated protein (MMP) confirmed ROS production, and cell arrest was also observed (Yu et al., 2019).

Nowadays, degenerative as well as non‐communicable diseases are prevalent in the world (Akhtar et al., 2023; Haroon et al., 2023; Wang & Hao, 2023). Due to increased drug resistance and relapse chances, plants are widely used to treat such diseases. They possess some bio‐medical properties due to bioactive compounds like MGF. This experiment was designed to explore different therapeutic activities, for example, antioxidant, antidiabetic, cytotoxicity of *Aquilaria crassna leaves* (ACE), and its bioactive compound MGF. To assess cytotoxic action, three cell lines, HepG2, HT‐29, and MDA‐MB‐231, of human hepatocellular carcinoma, colorectal carcinoma, and breast cancer. Results suggested that the cytotoxic potential of MGF was 67.85% for liver cancer, 63.52% for OC, and 62.93% for breast cancer at 100 μg/mL, which was a maximum concentration and also shows some free radical scavenging activity and antioxidant properties (Thitikornpong et al., 2019). The incidence of thyroid cancer is greater in women than men, while conjunctive therapy is used for its treatment due to its slow progression and poor differentiation. This experiment was conducted to investigate the anticancer and inhibitory action of MGF against thyroid cancer. Human thyroid cancer cell line TPC‐1 (papillary cell line) was cultivated in DMEM‐F12 (medium). Results showed that the functional capacity of TPC‐1 was reduced, and the number of cells became half after 24 h using two and four micromole concentrations of MGF. Downregulation of Bcl‐2, a protein that blocks apoptosis, and induction of Cas‐3, a protein that causes the degradation of DNA, showed the induction of the apoptotic pathways. Moreover, expressions of proapoptotic proteins, that is, Bid, Bad, and Bax, were also increased. Decreased levels of proliferating cell nuclear antigen (PCNA), an antigen involved in the repairing and replicating DNA, revealed the decreased progression and survival of cancerous cells (Zhang & Wang, 2018).

### 6.3 Overexpression of apoptotic proteins

Liver cancer is ranked fifth worldwide, with a mortality rate of over 9%. It is a silent cancer that spreads slowly and is also diagnosed at a later stage. The most familiar form of liver cancer is hepatocellular carcinoma (Nasrollahi et al., 2022; Yang et al., 2023). Some therapies (chemotherapy and radiotherapy) are commonly used to treat cancer but have a lot of toxic effects on cells other than cancer cells and also have side effects on the body. In this study, hepatocellular carcinoma was induced in Sprague–Dawley rats (immunodeficient rats having no NK, T, and B cells also vulnerable to infections) by using diethylnitrosamine (DEN, a carcinogen) in drinking water (0.01%) of rats for 12 weeks. After the 4 weeks of DEN, rats were treated with 50 mg MGF for 8 weeks. Histological examination of liver tissues and over‐expressed apoptotic proteins indicated that MGF inhibited cancer induction. MGF showed anticarcinogenic effects by altering oxidative stress in DEN‐induced carcinoma (Yang et al., 2019).

### 6.4 Inhibition of phosphatidylinositol 3‐kinase/protein kinase B (PI3K/Akt) pathways

Gastric carcinoma, the type of cancer affecting the stomach's epithelial lining, is commonly found in both men and women (Du et al., 2018). The most favorable factors which can decrease its incidence are eating a healthy diet, less smoking in males, and decreased rate of *H. pylori* infection (Carioli et al., 2020). At the initial stage of cancer, laparoscopic gastrectomy is considered the most effective treatment of gastric cancer (Katai et al., 2019, 2020; Kim et al., 2019; Song et al., 2023). This study conducted a 3‐(4,5‐dimethylthiazol‐2‐yl)‐2,5‐diphenyl‐2H‐tetrazolium bromide (MTT) assay to investigate the effects of MGF on cell functionality and progression. Two cell lines of gastric lining, SGC‐7901 and BCG‐823, were used to check their apoptotic rates by investigating the expressions of proteins related to apoptosis (a planned cell death). Results indicated that MGF repressed the cellular growth of both cells (SGC‐7901 and BCG‐823) in a time‐ and dose‐dependent way. The IC50 values were gradually decreased from 16 to 4.79 μ mole/liter for SGC‐7901 cells which were manifested to MGF for 24, 48, and 72 h. MGF caused a decrease in the expressions of Bcl‐2, B‐cell lymphoma‐extra‐large (Bcl‐xL) and induced myeloid leukemia cell differentiation protein (Mcl‐1) and increased expressions of Bad, Bax along with caspase‐3 and 9 in SGC‐7901 cells. This research concludes that MFG decreases the growth, progression, and new blood vessel formation in gastric cell lines by inhibiting PI3K/Akt pathways. So, MGF can safely treat stomach cancer as a chemotherapeutic agent (Du et al., 2018).

## 7 MODULATION OF IMMUNE RESPONSES

Prostate cancer is the most prevalent cancer among males, causing mortality. In contrast, castration‐resistant prostate cancer, commonly named CRPC, is a type of prostate cancer that also proliferates at low levels of testosterone hormone (the primary sex hormone of male) (Sekhoacha et al., 2022). Castration is when the testicle gland cannot produce a normal testosterone level. The tumor microenvironment plays a vital role in the function of immunity. In this experiment, MGF‐AuNPs were utilized by tumor‐containing mice to check their immunomodulation and effects on prostate cancer. Results showed that these nanoparticles had increased IL‐12 and TNF‐α (antitumor chemicals) levels with a simultaneous decrease in IL‐6 and IL‐10 (pre‐tumor cytokines). Observations indicated that IL‐12 increased 10 times, TNF‐α increased 10 times, and tumor‐supporting cytokines decreased by 2 times. MGF‐AuNPs showed no toxicity toward non‐cancerous cells due to less density of laminin receptors in normal cells but increased toxicity to cancerous cells due to overexpression of laminin receptors. MGF targeted splenic macrophages (which perform a phagocytic activity in the blood) by targeting the NFkB signaling pathway, which makes the connection between danger signals and pathogenic signals of a cell. There was inhibition of angiogenesis near cancer cells, which resulted in cell death. In the end, the tumor growth stopped, and a reduction in tumor volume also occurred (Khoobchandani et al., 2021; Pritam et al., 2021; Zhu & Dong, 2023).

Leukemia is a disease of blood‐making tissues like lymphocytes and bone marrow, causing less production of blood RBCs, WBCs, and coagulating cells (plate cells). Acute myeloid leukemia (AML) is a disease of myeloid cells, self‐regenerating, and immature cells that transform into monocytes (granulocytes). In AML, leukocytes (white blood cells) are produced less in fighting against invaders. Chemotherapy and radiotherapy were considered perfect treatments for leukemia, but they have some adverse effects on normal cells. This research aimed to assess the effects of MGF on weight gain and the immune system. The WEHI‐3 cell line was transferred in male mice, while doses of MGF were 40, 80, and 120 mg/kg body weight, respectively. Results suggested a subsequent increase in body weights, but decreased liver and spleen weights (spleen and liver became larger in leukemia) were also noticed. Increased CD3 T‐cell and CD19 B cells were seen, but CD11b monocyte and Mac‐3 macrophages (markers of macrophages) were reduced. Moreover, decreased phagocytosis of macrophages by peripheral blood mononuclear cells (PBMC). Splenocytes (WBCs of the spleen) were used to check the cytotoxic activity of NK cells and MGF, which did not affect the progression of T and B lymphocytes. The survival rate of leukemia mice depended upon the given dose of MGF, which was increased at a high dose of MGF (Shang et al., 2021).

Melanoma is one of the most dangerous and deadliest forms of skin cancer, having metastatic characteristics, a condition in which the primary tumor loses its cells that make a new tumor. Its treatments have not increased the survival rates of patients. This study was designed to evaluate the antiangiogenic effects of MGF and explore the hidden mechanism of action. Two cell lines of human A549 (epithelial line) and EA.hy 926 hybridoma (umbilical vein line) were used. Results revealed that MGF has anti‐inflammatory effects and interacts with lipid and calcium pathways to inhibit the release of chemicals through NFkB. Matrix metalloprotease 19, placental growth factor (part of VEGF), and vascular endothelial growth factor receptor 2 play a crucial role in forming new blood vessels (angiogenesis), and MGF inhibited migration of cancerous cells. IL‐6 (involved in inflammation), INF‐γ (cytokine), urokinase (convert plasminogen into plasmin), and CCL2 (monocyte chemoattractant) were also restricted to the site of the tumor as well as decreased the process of metastasis. All these actions hindered the formation of the new capillary tube for angiogenesis in human blood vessels of the placenta (in vitro) and a chicken's egg (in vivo). Thus, the described experimental research has explained the inhibition of angiogenesis by modulation of immune cells (Delgado‐Hernández et al., 2020).

## 8 MGF IN COMBINATION WITH OTHER CHEMOTHERAPEUTIC DRUGS

OC is a major gynecological problem, and evidence‐based studies suggest that YAP facilitates tumor formation (He et al., 2019). The incidence rate of OC is higher in Europe and North America than in Africa (Cabasag et al., 2022). Oral contraceptive pills can decrease the incidence of OC (Webb et al., 2017). Moreover, menopausal hormone therapy is directly linked with OC, but its use has decreased (Simin et al., 2020). In this experiment, human ovarian cells, OVCAR8, were introduced to female mice (*N* = 60) weighing 16–18 g. MGF + cisplatin (50 mg and 10 mg/kg BW) was the main phytochemical to investigate its effects on tumor volume. Results indicated that conjunctive therapy (MGF + Cisplatin) decreases tumor size (volume), increases mice's body weights, increases life span, and enhances the sensitivity of tumor cells to cisplatin. Therefore, combined with MGF, cisplatin can treat tumors resistant to cisplatin (He et al., 2019). Table 3 explains the “anticancer action of MGF based on animal studies” to check the efficiency of MGF in handling cancers and depicts its mechanism of action for different cancers.

**TABLE 3:** Anticancer action of MGF based on animal studies.

Cancer	MFG formulation	Cell line	Mechanism of action	Dose	Sample size	Findings/Results	References
Colorectal cancer	Soaking extract of MGF from bay, leaves, and stalk	CT26. WT adenocarcinoma of the colon of mice	By generating minor transcriptional changes in mitochondrial metabolic strain	100 mg	50	↓Tumor size up to 75%	Rodriguez‐Gonzalez et al. (2021)
Prostate cancer	(MGF‐AuNPs)	PC‐3 (prostate cancer) human cell line	By ↑levels of IL‐12 and TNF‐α and ↓levels of IL‐10 and IL‐6	0.5, 1, and 1.5 mg/kg BW	28	Inhibition of angiogenesis, ↓tumor volume and progression, induction of cell death	Khoobchandani et al. (2021)
Oral cancer	MGF held over corn oil + DMBA 0.5%	Oral squamous cell carcinoma of Hamster	By expelling metabolites of malicious cells	50 mg/kg B W	24	↑BW, no tumor formation, clear epithelium, normal level of LPO in plasma and cheek	Liu et al. (2021)
Triple‐negative breast cancer	MGF extract from greenery, bay, and seed coat	MDA‐MB231 Human TNBC cell	By suppressing 3 binding sites of PKM2 (PDB ID: 6V74)		51	Suppression of PKM2 pathway, negative impact on TNBC growth	Rasul et al. (2021)
Leukemia (Blood cancer)	MGF	WEHI‐3 myelomonocytic leukemia cell line of BALB/c mice	↓phagocytosis of macrophages by peripheral blood mononuclear cells (PBMC)	120 mg/kg of MGF in DMSO	80	↑BW, ↓spleen and liver weights, ↑survival rate	Shang et al. (2021)
Lung cancer	MGF + LPS	Human NSCLC cell line A549 (CCL‐185), NCI‐H460 (HTB‐177), and squamous cell carcinoma cell line NCI‐H520 (HTB‐182)	By activation of caspase‐8	800 μg/mL	NA	Least progression of tumor cells, suppression of CXCR4, reversed expressions of PER 1, NLRP3, and excretion of settled IL‐1β	Lin et al. (2020)
Ovarian cancer	MGF Powder	A2780(ovarian adenocarcinoma), ES‐2 (ovarian clear cell carcinoma	By ↓expressions of matrix metalloproteinase 2 as well as matrix metalloproteinase 9	60 mg/kg B W	NA	Suppressed progression of cancer, ↓ cell motility, and migration, inhibition of tumor growth	Zeng et al. (2020)
Bronchial cancer	MGF from aqueous extract of bark	B16 melanoma cells (B16F10 cell line), EA. hy 926 hybridoma cell line of the human umbilical vein endothelial cell	By inhibiting calcium signaling, metabolism of lipid and inflammatory pathways (NFkB, HMGB1, NO)	240 μM	NA	Inhibition of cell movement, no cytotoxic effects on normal cells, inhibition of endothelial growth, inhibition of capillary growth, and development of tube in placental blood vessel	Delgado‐Hernández et al. (2020)
Ovarian cancer	MGF + Cisplatin	Human ovarian adenocarcinoma OVCAR8 cells were injected in BALB/c nude female mice	Through regulation (suppression) of the YAP pathway	50 mg/kg BW + 10 mg/kg B W	60	↓tumor size, MGF increases sensitivity of the ovarian tumor to cisplatin, ↑BW, and survival rate	He et al. (2019)
Prostate cancer	MGF‐198AuNPs (MGF from mango peel)	Prostate tumor (PC‐3) xenografts implanted in SCID mice	Due to the higher metabolism of xanthone (glucose) in tumor cells	1.55 mg	5	Destruction of the main prostate tumors, stopped tumor migration, the retention time for 90% of nanoparticles was 24 hours	Katti et al. (2018)
Hepatocellular carcinoma	MGF	Human HCC, cell line inoculating, luciferase‐tagged MHCC97L BALB/c‐nu/nu mouse	By downregulation of the LEF1 gene in the Wnt signaling pathway	50 mg/kg B W	244	No toxic effect of MGF on mice, ↓tumor size, or non‐hostile growth with a border between hepatic and tumor tissues	Tan et al. (2018)
Liver cancer	MGF	The hepatic cell line of SD rats	Overexpression of apoptotic proteins	50 mg	N/A	Inhibition of cancer	Tempero et al. (2019)
Mammary Carcinoma	MGF	Mammary cell line	↑levels of antioxidant enzymes, inhibition of NFkB, upregulation of Bcl‐2	100 mg/kg B W	N/A	↓ carcinogenesis	Xin Wang et al. (2021)

Honeybush, called *C. intermedia*, is a shrub that contains MGF and other phytochemicals used to treat cancers. MGF from *M. indica*, L. is a therapeutic agent used in cancer therapy. Previous studies showed that MGF action augmented with other chemotherapeutic drugs like DOX and Ayurvedic. In this experiment, researchers used gold‐coated Honeybush nanoparticles (HB‐AuNPs) and MGF‐AuNPs separately and combined them with DOX to check their impact on cancerous and non‐cancerous cells. Three types of cell lines, colon cell line (Caco‐2), prostate cell line (PC‐3), glioblastoma cell line (U87), cancer, and normal breast cell line (MCF‐12A), were used in in vitro assays. Results suggested that the efficiency of breast cells (non‐cancerous) was unaffected by both nanoparticles, even at their highest concentration. Colon cells' toxicity was less than brain and prostate cancer cell lines. Both particles were stable in water. Both nanoparticles were tested with DOX in cancerous colon cells, and results showed that the efficiency of cells decreased from 40% to 70% and toxicity was greater at higher concentrations. So, MGF‐AuNPs and HB‐AuNPs can be synergistically used with DOX and may give better results in treating cancers (Aboyewa et al., 2021).

## 9 CONCLUSIONS

Cancer is a major cause of mortality. While most cancers are asymptomatic and diagnosed at a later stage, some cancers are resistant to chemotherapy, radiotherapy, or other drugs. Novel studies show that MGF can treat various cancers with no or lesser side effects on normal cells. MGF inhibits tumor growth, proliferation, motility, and angiogenesis and increases apoptosis by targeting interleukins, growth factors, genes, enzymes, and signaling pathways. MGF can also be used with other therapeutic drugs to increase their sensitivity to the body. It can also be used instead of drugs for which tumors show resistance. MGF is safe, but its dose should differ according to body weight for different cancers. Most of the experiments in this scenario were in vivo (using different animals like mice and hamsters) and in vitro (using different human cell lines). However, due to its low bioavailability, MGF is not directly used for cancer patients. All the literature included in this review only describes animal trials and in vitro results. It shows positive effects for animals, but trials for humans are needed. Previous literature revealed that pure MGF has low solubility in water, less bioavailability, and the least distribution to cancerous cells. Scientific data regarding its effective dose for greater cytotoxic effects still need to be included. Certain techniques are available to increase its rate of distribution and bioavailability, like nanoparticles or combining with other drugs, but more is needed for its clinical use. The available scientific literature must provide more surety for its direct use in humans. Except for all the above discussions, further research is needed to unveil hidden aspects of MGF for its clinical use.

## AUTHOR CONTRIBUTIONS

**Humaira Iqbal:** Data curation (equal); formal analysis (equal); funding acquisition (equal); investigation (equal); methodology (equal); resources (equal); software (equal); supervision (equal); validation (equal); visualization (equal); writing – original draft (equal). **Muhammad Inam‐Ur‐Raheem:** Investigation (equal); methodology (equal); project administration (equal); resources (equal); software (equal); writing – original draft (equal). **Seemal Munir:** Formal analysis (equal); funding acquisition (equal); investigation (equal); methodology (equal); project administration (equal); validation (equal); visualization (equal). **Roshina Rabail:** Data curation (equal); formal analysis (equal); funding acquisition (equal); resources (equal); software (equal); supervision (equal); validation (equal); visualization (equal); writing – original draft (equal). **Sadia Kafeel:** Conceptualization (equal); data curation (equal); formal analysis (equal); software (equal); validation (equal); visualization (equal). **Arashi Shahid:** Data curation (equal); formal analysis (equal); resources (equal); software (equal); supervision (equal); validation (equal); visualization (equal); writing – original draft (equal). **Amin Mousavi Khaneghah:** Funding acquisition (equal); project administration (equal); resources (equal); software (equal); supervision (equal); visualization (equal); writing – original draft (equal); writing – review and editing (equal). **Rana Muhammad Aadil:** Data curation (equal); funding acquisition (equal); methodology (equal); project administration (equal); resources (equal); software (equal); supervision (equal); visualization (equal); writing – original draft (equal); writing – review and editing (equal).

## CONFLICT OF INTEREST STATEMENT

None.

## Data Availability

The data will be available on request from the corresponding authors.

## References

[fsn33869-bib-0001] Aboyewa, J. A. , Sibuyi, N. R. , Meyer, M. , & Oguntibeju, O. O. (2021). Gold nanoparticles synthesized using extracts of cyclopia intermedia, commonly known as honeybush, amplify the cytotoxic effects of doxorubicin. Nanomaterials, 11(1), 132. 33429945 10.3390/nano11010132 PMC7826697

[fsn33869-bib-0108] Adoho, A. C. C. , Konmy, B. B. S. , Olounladé, P. A. , Azando, E. V. , Hounzangbé‐Adoté, M. S. , & Gbangboché, B. A. (2022). Phytochemistry and larval toxicity of *Ipomea asarifolia*, *Commelina diffusa*, *Acalypha ciliata* and *Eleusine indica* against *Artemia salina* . International Journal of Veterinary Science, 11(2), 121–128.

[fsn33869-bib-0102] Akhtar, T. , Shahid, S. , Asghar, A. , Naeem, M. I. , Aziz, S. , & Ameer, T. (2023). Utilisation of herbal bullets against Newcastle disease in poultry sector of Asia and Africa (2012–2022). International Journal of Agriculture and Biosciences, 12(1), 56–65.

[fsn33869-bib-0002] AlMasri, S. , Zenati, M. , Hammad, A. , Nassour, I. , Liu, H. , Hogg, M. E. , Zeh, H. J. , Boone, B. , Bahary, N. , Singhi, A. D. , Lee, K. K. , Paniccia, A. , & Zureikat, A. H. (2022). Adaptive dynamic therapy and survivorship for operable pancreatic cancer. JAMA Network Open, 5(6), e2218355. 35737385 10.1001/jamanetworkopen.2022.18355 PMC9227002

[fsn33869-bib-0003] Balasar, M. , Özkent, M. S. , Aydin, A. , Taskapu, H. H. , Atici, A. , Ecer, G. , & Sonmez, M. G. (2022). The benign renal masses that were exposed after nephron‐sparing surgery: “postsurgical fatty tumor.” Is it related to the surgical technique? Journal of Kidney Cancer and VHL, 9(1), 1. 10.15586/jkcvhl.v9i1.195 PMC8571989 34888127

[fsn33869-bib-0004] Bhattacharyya, S. , Ahmmed, S. M. , Saha, B. P. , & Mukherjee, P. K. (2014). Soya phospholipid complex of mangiferin enhances its hepatoprotectivity by improving its bioavailability and pharmacokinetics. Journal of the Science of Food and Agriculture, 94(7), 1380–1388. 24114670 10.1002/jsfa.6422

[fsn33869-bib-0005] Bray, F. , Ferlay, J. , Soerjomataram, I. , Siegel, R. L. , Torre, L. A. , & Jemal, A. (2018). Global cancer statistics 2018: GLOBOCAN estimates of incidence and mortality worldwide for 36 cancers in 185 countries. CA: A Cancer Journal for Clinicians, 68(6), 394–424. 30207593 10.3322/caac.21492

[fsn33869-bib-0006] Buhari, G. K. , Kalkan, İ. K. , Ateş, H. , Bahçecioğlu, S. N. , Demir, Ş. , Yeşilkaya, S. , Solak, G. T. V. , Aksu, K. , & Erkekol, F. Ö. (2023). Platin desensitizations in thoracic malignancies and risk factors for breakthrough reactions. Allergologia et Immunopathologia, 51(2), 130–136. 36916098 10.15586/aei.v51i2.779

[fsn33869-bib-0007] Cabasag, C. J. , Fagan, P. J. , Ferlay, J. , Vignat, J. , Laversanne, M. , Liu, L. , van der Aa, M. A. , Bray, F. , & Soerjomataram, I. (2022). Ovarian cancer today and tomorrow: A global assessment by world region and Human Development Index using GLOBOCAN 2020. International Journal of Cancer, 151, 1535–1541. 35322413 10.1002/ijc.34002

[fsn33869-bib-0008] Cao, C. , Friedenreich, C. M. , & Yang, L. (2022). Association of daily sitting time and leisure‐time physical activity with survival among US cancer survivors. JAMA Oncology, 8(3), 395–403. 34989765 10.1001/jamaoncol.2021.6590 PMC8739832

[fsn33869-bib-0009] Carioli, G. , Bertuccio, P. , Boffetta, P. , Levi, F. , La Vecchia, C. , Negri, E. , & Malvezzi, M. (2020). European cancer mortality predictions for the year 2020 with a focus on prostate cancer. Annals of Oncology, 31(5), 650–658. 32321669 10.1016/j.annonc.2020.02.009

[fsn33869-bib-0010] Carminatti, T. , Marchiñena, P. A. G. , González, I. P. T. , de Miguel, V. , Serra, M. M. , Kalfayan, P. G. , & Jurado, A. M. (2023). Hereditary renal cell carcinoma: Is age an independent criterion for genetic testing? A large cohort from a Latin America referral center. Journal of Kidney Cancer and VHL, 10(3), 17–22. 37555194 10.15586/jkcvhl.v10i3.242 PMC10404984

[fsn33869-bib-0011] Chen, M. , Zhong, L. , Zhang, Z. , Peng, C. , Ke, D. , Gan, P. , Wang, Z. , Wei, R. , Liu, W. , & Yang, J. (2022). Isolation and identification of *Colletotrichum* as fungal pathogen from tea and preliminary fungicide screening. Quality Assurance and Safety of Crops & Foods, 14(1), 92–101.

[fsn33869-bib-0012] Choi, W. S. W. , Boland, J. , & Lin, J. (2021). Hypoxia‐inducible factor‐2α as a novel target in renal cell carcinoma. Journal of Kidney Cancer and VHL, 8(2), 1–7. 10.15586/jkcvhl.v8i1.170 PMC8033537 33868900

[fsn33869-bib-0013] Coco, D. , & Leanza, S. (2022). Von Hippel‐Lindau syndrome: Medical syndrome or surgical syndrome? A surgical perspective. Journal of Kidney Cancer and VHL, 9(1), 27–32. 10.15586/jkcvhl.v9i1.206 PMC8652351 34963877

[fsn33869-bib-0014] Conroy, T. , Hammel, P. , Hebbar, M. , Ben Abdelghani, M. , Wei, A. , Raoul, J. , Choné, L. , Francois, E. , Artru, P. , Biagi, J. J. , Lecomte, T. , Assenat, E. , Faroux, R. , Ychou, M. , Volet, J. , Sauvanet, A. , Breysacher, G. , Di Fiore, F. , Cripps, C. , … Canadian Cancer Trials Group and the Unicancer‐GI–PRODIGE Group . (2018). FOLFIRINOX or gemcitabine as adjuvant therapy for pancreatic cancer. The New England Journal of Medicine, 379, 2395–2406. 30575490 10.1056/NEJMoa1809775

[fsn33869-bib-0015] Culp, M. B. , Soerjomataram, I. , Efstathiou, J. A. , Bray, F. , & Jemal, A. (2020). Recent global patterns in prostate cancer incidence and mortality rates. European Urology, 77(1), 38–52. 31493960 10.1016/j.eururo.2019.08.005

[fsn33869-bib-0016] Dai, J.‐N. , Liu, B.‐L. , Ji, D. , Yuan, L. , Zhou, W.‐Y. , & Li, H.‐X. (2023). Extraction, isolation, identification, and bioactivity of polysaccharides from *Antrodia cinnamomea* . Quality Assurance and Safety of Crops & Foods, 15(4), 60–76.

[fsn33869-bib-0018] Djiwa, T. , Sabi, K. A. , Simgban, P. , Bombonne, M. , Sama, B. M. , Tchaou, M. , & Darré, T. (2022). Renal leiomyosarcoma, a rare presentation. Journal of Kidney Cancer and VHL, 9(1), 51–54. 35433232 10.15586/jkcvhl.v9i1.216 PMC8953749

[fsn33869-bib-0019] Drobner, J. , Portal, D. , Runcie, K. , Yang, Y. , & Singer, E. A. (2023). Systemic treatment for advanced and metastatic non‐clear cell renal cell carcinoma: Examining modern therapeutic strategies for a notoriously challenging malignancy. Journal of Kidney Cancer and VHL, 10(3), 37–60. 37789902 10.15586/jkcvhl.v10i3.295 PMC10542704

[fsn33869-bib-0020] Du, M. , Wen, G. , Jin, J. , Chen, Y. , Cao, J. , & Xu, A. (2018). Mangiferin prevents the growth of gastric carcinoma by blocking the PI3K‐Akt signalling pathway. Anti‐Cancer Drugs, 29(2), 167–175. 29215373 10.1097/CAD.0000000000000583

[fsn33869-bib-0106] El‐Dawy, K. , Mohamed, D. , & Abdou, Z. (2022). Nanoformulations of pentacyclic triterpenoids: Chemoprevention and anticancer. International Journal of Veterinary Science, 11, 384–391.

[fsn33869-bib-0105] Elsayed, A. , Elkomy, A. , Alkafafy, M. , Elkammar, R. , Fadl, S. E. , Abdelhiee, E. Y. , Abdeen, A. , Youssef, G. , Shaheen, H. , Soliman, A. , & Aboubakr, M. (2022). Ameliorating effect of lycopene and N‐acetylcysteine against cisplatin‐induced cardiac injury in rats. Pakistan Veterinary Journal, 42, 107–111.

[fsn33869-bib-0021] Fei, B. , Dai, W. , & Zhao, S. (2018). Efficacy, safety, and cost of therapy of the traditional Chinese medicine, catalpol, in patients following surgical resection for locally advanced colon cancer. Medical Science Monitor: International Medical Journal of Experimental and Clinical Research, 24, 3184–3192. 29763415 10.12659/MSM.907569 PMC5975072

[fsn33869-bib-0022] Ferlay, J. , Colombet, M. , Soerjomataram, I. , Parkin, D. M. , Piñeros, M. , Znaor, A. , & Bray, F. (2021). Cancer statistics for the year 2020: An overview. International Journal of Cancer, 149(4), 778–789. 10.1002/ijc.33588 33818764

[fsn33869-bib-0023] Giaquinto, A. N. , Miller, K. D. , Tossas, K. Y. , Winn, R. A. , Jemal, A. , & Siegel, R. L. (2022). Cancer statistics for African American/Black People 2022. CA: A Cancer Journal for Clinicians, 72(3), 202–229. 35143040 10.3322/caac.21718

[fsn33869-bib-0024] Giaquinto, A. N. , Sung, H. , Miller, K. D. , Kramer, J. L. , Newman, L. A. , Minihan, A. , Jemal, A. , & Siegel, R. L. (2022). Breast cancer statistics, 2022. CA: A Cancer Journal for Clinicians, 72(6), 524–541. 36190501 10.3322/caac.21754

[fsn33869-bib-0025] Grauzdytė, D. , Raudoniūtė, J. , Kulvinskienė, I. , Bagdonas, E. , Stasiulaitienė, I. , Martuzevičius, D. , Bironaitė, D. , Aldonytė, R. , & Venskutonis, P. R. (2019). Cytoprotective effects of mangiferin and Z‐ligustilide in PAH‐exposed human airway epithelium in vitro. Nutrients, 11(2), 218. 30678167 10.3390/nu11020218 PMC6412222

[fsn33869-bib-0026] Guo, H. , Chen, M. , Li, M. , Hu, M. , Chen, B. , & Zhou, C. (2019). Pharmacokinetic comparisons of mangiferin and mangiferin monosodium salt in rat plasma by UPLC‐MS/MS. Journal of Chemistry, 2019, 1–12.

[fsn33869-bib-0103] Haroon, M. , Anas, M. , Naurin, I. , Afzal, R. , Irfan, U. , Tariq, H. , Idrees, F. , Taj, M. H. , & Rukh, M. (2023). Autoimmunity in plants; A powerful weapon in kingdom plantae to combat stresses. International Journal of Agriculture and Biosciences, 12(3), 159–164.

[fsn33869-bib-0027] He, W. , You, Y. , Du, S. , Lei, T. , Wang, H. , Li, X. , He, X. , Tong, R. , & Wang, Y. (2019). Anti‐neoplastic effect of mangiferin on human ovarian adenocarcinoma OVCAR8 cells via the regulation of YAP. Oncology Letters, 17(1), 1008–1018. 30655860 10.3892/ol.2018.9708 PMC6313056

[fsn33869-bib-0028] Hou, C. , Sun, F. , Liang, Y. , Nasab, E. M. , & Athari, S. S. (2023). Effect of transduced mesenchymal stem cells with IL‐10 gene on control of allergic asthma. Allergologia et Immunopathologia, 51(2), 45–51. 10.15586/aei.v51i2.789 36916087

[fsn33869-bib-0029] Hu, Y. , Xiang, X. , Zhang, Y. , Tian, Z. , & Wang, L. (2022). Aloin promotes oral squamous cell carcinoma cell apoptosis and autophagy through Akt/mTOR pathway. Quality Assurance and Safety of Crops & Foods, 14(2), 58–65.

[fsn33869-bib-0030] Hung, L.‐C. , Kung, P.‐T. , Lung, C.‐H. , Tsai, M.‐H. , Liu, S.‐A. , Chiu, L.‐T. , Huang, K. H. , & Tsai, W.‐C. (2020). Assessment of the risk of oral cancer incidence in a high‐risk population and establishment of a predictive model for oral cancer incidence using a population‐based cohort in Taiwan. International Journal of Environmental Research and Public Health, 17(2), 665. 31968579 10.3390/ijerph17020665 PMC7014279

[fsn33869-bib-0109] Ijaz, M. U. , Rafi, Z. , Hamza, A. , Tariq, M. , Alkahtani, S. , Alkahtane, A. A. , & Riaz, M. N. (2023). Tectochrysin attenuates cisplatin‐induced hepatotoxicity by restoring biochemical, inflammatory and histological profile in rats. Pakistan Veterinary Journal, 43(2), 366–370.

[fsn33869-bib-0031] Ishibashi, C. M. , de Oliveira, C. E. C. , Guembarovski, R. L. , Hirata, B. K. B. , Vitiello, G. A. F. , Guembarovski, A. L. , Amarante, M. K. , de Oliveira, K. B. , Kishima, M. O. , Ariza, C. B. , & Watanabe, M. A. E. (2021). Genetic polymorphisms of the TGFB1 signal peptide and promoter region: Role in Wilms tumor susceptibility? Journal of Kidney Cancer and VHL, 8(4), 22–31. 34722128 10.15586/jkcvhl.v8i4.182 PMC8532353

[fsn33869-bib-0032] Islami, F. , Goding Sauer, A. , Miller, K. D. , Siegel, R. L. , Fedewa, S. A. , Jacobs, E. J. , Jacobs, E. J. , McCullough, M. L. , Patel, A. V. , Ma, J. , Soerjomataram, I. , Flanders, W. D. , Brawley, O. W. , Gapstur, S. M. , & Jemal, A. (2018). Proportion and number of cancer cases and deaths attributable to potentially modifiable risk factors in the United States. CA: A Cancer Journal for Clinicians, 68(1), 31–54. 29160902 10.3322/caac.21440

[fsn33869-bib-0033] Jabeen, A. , Malik, G. , Mir, J. I. , & Rasool, R. (2023). Nutrigenomics: Linking food to genome. Italian Journal of Food Science, 35(1), 26–40.

[fsn33869-bib-0034] Jiang, M. , & Zheng, S. (2022). Geniposide inhibits non‐small cell lung cancer cell migration and angiogenesis by regulating PPARγ/VEGF‐A pathway. Quality Assurance and Safety of Crops & Foods, 14(1), 46–54.

[fsn33869-bib-0035] Kari, A. , Zhihua, M. , Aili, Z. , Adili, A. , Hairula, N. , & Abuduhaer, A. (2022). Knockdown of EPSTI1 alleviates lipopolysaccharide‐induced inflammatory injury through regulation of NF‐κB signaling in a cellular pneumonia model. Allergologia et Immunopathologia, 50(3), 106–112. 35527663 10.15586/aei.v50i3.581

[fsn33869-bib-0036] Katai, H. , Mizusawa, J. , Katayama, H. , Kunisaki, C. , Sakuramoto, S. , Inaki, N. , Kinoshita, T. , Iwasaki, Y. , Misawa, K. , Takiguchi, N. , Kaji, M. , Okitsu, H. , Yoshikawa, T. , & Terashima, M. (2019). Single‐arm confirmatory trial of laparoscopy‐assisted total or proximal gastrectomy with nodal dissection for clinical stage I gastric cancer: Japan Clinical Oncology Group study JCOG1401. Gastric Cancer, 22, 999–1008. 30788750 10.1007/s10120-019-00929-9

[fsn33869-bib-0037] Katai, H. , Mizusawa, J. , Katayama, H. , Morita, S. , Yamada, T. , Bando, E. , Ito, S. , Takagi, M. , Takagane, A. , Teshima, S. , Koeda, K. , Nunobe, S. , Yoshikawa, T. , Terashima, M. , & Sasako, M. (2020). Survival outcomes after laparoscopy‐assisted distal gastrectomy versus open distal gastrectomy with nodal dissection for clinical stage IA or IB gastric cancer (JCOG0912): A multicentre, non‐inferiority, phase 3 randomised controlled trial. The Lancet Gastroenterology & Hepatology, 5(2), 142–151. 31757656 10.1016/S2468-1253(19)30332-2

[fsn33869-bib-0038] Katti, K. V. , Khoobchandani, M. , Thipe, V. C. , Al‐Yasiri, A. Y. , Katti, K. K. , Loyalka, S. K. , Sakr, T. M. , & Lugão, A. B. (2018). Prostate tumor therapy advances in nuclear medicine: Green nanotechnology toward the design of tumor specific radioactive gold nanoparticles. Journal of Radioanalytical and Nuclear Chemistry, 318(3), 1737–1747.

[fsn33869-bib-0039] Khoobchandani, M. , Khan, A. , Katti, K. K. , Thipe, V. C. , Al‐Yasiri, A. Y. , MohanDoss, D. K. , Nicholl, M. B. , Lugão, A. B. , Hans, C. P. , & Katti, K. V. (2021). Green nanotechnology of MGF‐AuNPs for immunomodulatory intervention in prostate cancer therapy. Scientific Reports, 11(1), 1–30. 34408231 10.1038/s41598-021-96224-8 PMC8373987

[fsn33869-bib-0040] Kim, H. H. , Han, S. U. , Kim, M. C. , Kim, W. , Lee, H. J. , Ryu, S. W. , Cho, S. , Kim, C. Y. , Yang, H. K. , Park, D. J. , Song, K. Y. , Lee, S. I. , Ryu, S. Y. , Lee, J. H. , & Hyung, W. J. (2019). Effect of laparoscopic distal gastrectomy vs open distal gastrectomy on long‐term survival among patients with stage I gastric cancer: the KLASS‐01 randomized clinical trial. JAMA Oncology, 5(4), 506–513. 30730546 10.1001/jamaoncol.2018.6727 PMC6459124

[fsn33869-bib-0041] Kimura, T. , & Egawa, S. (2018). Epidemiology of prostate cancer in Asian countries. International Journal of Urology, 25(6), 524–531. 29740894 10.1111/iju.13593

[fsn33869-bib-0112] Krishnaveni, P. , Thangapandiyan, M. , Raja, P. , & Rao, G. V. S. (2023). Pathological and molecular studies on antitumor effect of curcumin and curcumin solid lipid nanoparticles. Pakistan Veterinary Journal, 43, 315–320.

[fsn33869-bib-0042] Leopold, Z. , Passarelli, R. , Mikhail, M. , Tabakin, A. , Chua, K. , Ennis, R. D. , Nosher, J. , & Singer, E. A. (2022). Modern management of localized renal cell carcinoma—Is ablation part of the equation? Journal of Kidney Cancer and VHL, 9(3), 5–23. 36060450 10.15586/jkcvhl.v9i3.233 PMC9396960

[fsn33869-bib-0043] Li, J. , Cao, L. , Yuan, C. , Jiang, Z. , Cai, H. , Xu, W. , Han, Y. , Chen, L. , Zhang, Q. , Jiang, R. , & Liu, J. (2023). *Ganoderma lucidum* extract reverses hepatocellular carcinoma multidrug resistance via inhibiting the function of P‐glycoprotein in vitro and in vivo. Italian Journal of Food Science, 35(3), 90–98.

[fsn33869-bib-0044] Lin, Y. S. , Tsai, K. L. , Chen, J. N. , & Wu, C. S. (2020). Mangiferin inhibits lipopolysaccharide‐induced epithelial‐mesenchymal transition (EMT) and enhances the expression of tumor suppressor gene PER1 in non‐small cell lung cancer cells. Environmental Toxicology, 35(10), 1070–1081. 32420661 10.1002/tox.22943

[fsn33869-bib-0045] Liu, J. , Xu, F. , Cheng, W. , & Gao, L. (2020). Identification and verification of a ten‐gene signature predicting overall survival for ovarian cancer. Experimental Cell Research, 395(2), 112235. 32805252 10.1016/j.yexcr.2020.112235

[fsn33869-bib-0046] Liu, L. , Erickson, N. T. , Ricard, I. , von Weikersthal, L. F. , Lerch, M. M. , Decker, T. , Kiani, A. , Kaiser, F. , Heintges, T. , Kahl, C. , Kullmann, F. , Scheithauer, W. , Link, H. , Höffkes, H. G. , Moehler, M. , Gesenhues, A. B. , Theurich, S. , Michl, M. , Modest, D. P. , … Holch, J. W. (2022). Early weight loss is an independent risk factor for shorter survival and increased side effects in patients with metastatic colorectal cancer undergoing first‐line treatment within the randomized phase III trial FIRE‐3 (AIO KRK‐0306). International Journal of Cancer, 150(1), 112–123. 34431518 10.1002/ijc.33775

[fsn33869-bib-0047] Liu, M. , Liu, Y. , Ge, Y. , Zhong, Z. , Wang, Z. , Wu, T. , Zhao, X. , & Zu, Y. (2020). Solubility, antioxidation, and oral bioavailability improvement of mangiferin microparticles prepared using the supercritical antisolvent method. Pharmaceutics, 12(2), 90. 31979138 10.3390/pharmaceutics12020090 PMC7076360

[fsn33869-bib-0048] Liu, M. , Wen, C. , & Pan, S. (2021). Modulator effect of mangiferin on biochemical characterization in 7, 12‐dimethylbenz [a] anthracene induced oral cancer in experimental hamsters. Veterinary Medicine and Science, 7(5), 2015–2025. 33949808 10.1002/vms3.500 PMC8464247

[fsn33869-bib-0049] Liu, R. , Liu, Z. , Zhang, C. , & Zhang, B. (2012). Nanostructured lipid carriers as novel ophthalmic delivery system for mangiferin: Improving in vivo ocular bioavailability. Journal of Pharmaceutical Sciences, 101(10), 3833–3844. 22767401 10.1002/jps.23251

[fsn33869-bib-0050] Liu, Z. , Peng, Q. , Li, Y. , & Gao, Y. (2018). Resveratrol enhances cisplatin‐induced apoptosis in human hepatoma cells via glutamine metabolism inhibition. BMB Reports, 51(9), 474–479. 30103844 10.5483/BMBRep.2018.51.9.114 PMC6177506

[fsn33869-bib-0051] Lo Galbo, V. , Lauricella, M. , Giuliano, M. , Emanuele, S. , Carlisi, D. , Calvaruso, G. , De Blasio, A. , & D'Anneo, A. (2021). Redox imbalance and mitochondrial release of apoptogenic factors at the forefront of the antitumor action of mango peel extract. Molecules, 26(14), 4328. 34299603 10.3390/molecules26144328 PMC8303932

[fsn33869-bib-0052] Ma, H. , Chen, H. , Sun, L. , Tong, L. , & Zhang, T. (2014). Improving permeability and oral absorption of mangiferin by phospholipid complexation. Fitoterapia, 93, 54–61. 24220727 10.1016/j.fitote.2013.10.016

[fsn33869-bib-0053] Malagón, T. , Yong, J. H. , Tope, P. , Miller, W. H., Jr. , Franco, E. L. , & McGill Task Force on the Impact of COVID‐19 on Cancer Control and Care . (2022). Predicted long‐term impact of COVID‐19 pandemic‐related care delays on cancer mortality in Canada. International Journal of Cancer, 150(8), 1244–1254. 34843106 10.1002/ijc.33884 PMC9015510

[fsn33869-bib-0054] Meng, L. , Gu, T. , Wang, J. , Zhang, H. , & Nan, C. (2023). Knockdown of PHLDA1 alleviates sepsis‐induced acute lung injury by downregulating NLRP3 inflammasome activation. Allergologia et Immunopathologia, 51(5), 41–47. 37695229 10.15586/aei.v51i5.940

[fsn33869-bib-0055] Miller, K. D. , Nogueira, L. , Devasia, T. , Mariotto, A. B. , Yabroff, K. R. , Jemal, A. , Kramer, J. , & Siegel, R. L. (2022). Cancer treatment and survivorship statistics, 2022. CA: A Cancer Journal for Clinicians, 72(5), 409–436. 35736631 10.3322/caac.21731

[fsn33869-bib-0056] Miller, K. D. , Nogueira, L. , Mariotto, A. B. , Rowland, J. H. , Yabroff, K. R. , Alfano, C. M. , Jemal, A. , Kramer, J. L. , & Siegel, R. L. (2019). Cancer treatment and survivorship statistics, 2019. CA: A Cancer Journal for Clinicians, 69(5), 363–385. 31184787 10.3322/caac.21565

[fsn33869-bib-0057] Miller, K. D. , Ortiz, A. P. , Pinheiro, P. S. , Bandi, P. , Minihan, A. , Fuchs, H. E. , Martinez Tyson, D. , Tortolero‐Luna, G. , Fedewa, S. A. , Jemal, A. M. , & Siegel, R. L. (2021). Cancer statistics for the US Hispanic/Latino population, 2021. CA: A Cancer Journal for Clinicians, 71(6), 466–487. 34545941 10.3322/caac.21695

[fsn33869-bib-0058] Mitsogiannis, I. C. , Mitsogianni, M. , Papathanassiou, M. , Anagnostou, M. , Tamposis, I. , Mitrakas, L. , Samara, M. , Tzortzis, V. , & Vlachostergios, P. J. (2022). Current options for second‐line systemic therapy in metastatic renal cell carcinoma. Journal of Kidney Cancer and VHL, 9(3), 29–40. 36310639 10.15586/jkcvhl.v9i3.243 PMC9551369

[fsn33869-bib-0101] Mubashir, A. , Ghani, A. , & Mubashar, A. (2022). Common medicinal plants effective in peptic ulcer treatment: A nutritional review. International Journal of Agriculture and Biosciences, 11(2), 70–74.

[fsn33869-bib-0059] Nabil, W. N. N. , Lim, R. J. , Chan, S. Y. , Lai, N. M. , & Liew, A. C.i. (2018). A systematic review on Chinese herbal treatment for radiotherapy‐induced xerostomia in head and neck cancer patients. Complementary Therapies in Clinical Practice, 30, 6–13. 29389481 10.1016/j.ctcp.2017.10.004

[fsn33869-bib-0060] Nasrollahi, H. , Eslahi, A. , Ariafar, A. , Ahmed, F. , & Monabati, A. (2022). Primary rhabdomyosarcoma of kidney with local recurrence and liver metastasis in adults: A case report. Journal of Kidney Cancer and VHL, 9(1), 55–58. 35529801 10.15586/jkcvhl.v9i1.218 PMC9021009

[fsn33869-bib-0062] Nurhadi, B. , Selly, S. , Nurhasanah, S. , Saputra, R. A. , & Arifin, H. R. (2022). The virgin coconut oil (VCO) emulsion powder characteristics: Effect of Pickering emulsion with microcrystalline cellulose (MCC) and different drying techniques. Italian Journal of Food Science, 34(1), 67–85.

[fsn33869-bib-0063] Othman, S. N. N. , & Seka, M. (2019). In‐vitro antioxidant and cytotoxic activities of silver nanoparticles of mangiferin isolated from *Mangifera indica* . Journal of Global Pharma Technology, 11(6), 10–15.

[fsn33869-bib-0064] Plonski, J. J. S. , Fernández‐Pello, S. , Jiménez, L. R. , Rodríguez, I. G. , Calvar, L. A. , & Villamil, L. R. (2021). Impact of body mass index on survival of metastatic renal cancer. Journal of Kidney Cancer and VHL, 8(2), 49–54. 34414066 10.15586/jkcvhl.v8i2.169 PMC8336599

[fsn33869-bib-0065] Pourzafar, Z. , Elhamirad, A. H. , Zenoozian, M. S. , & Armin, M. (2023). Optimization of producing functional sponge cake using a combination extract of green tea, white tea, and ginger. Italian Journal of Food Science, 35(2), 33–43.

[fsn33869-bib-0066] Prasad, V. , Sreelakshmi, C. V. , Chandran, K. R. , Agrawal, S. , Pooleri, G. K. , & Sao, A. (2023). Primary well‐differentiated neuroendocrine tumor of the kidney. Journal of Kidney Cancer and VHL, 10(2), 8. 37197692 10.15586/jkcvhl.v10i2.277 PMC10184039

[fsn33869-bib-0067] Pritam, P. , Manna, S. , Sahu, A. , Swain, S. S. , Ramchandani, S. , Bissoyi, S. , Panda, M. K. , Sing, Y. D. , Mohanta, Y. K. , Behera, R. K. , & Jit, B. P. (2021). Eosinophil: A central player in modulating pathological complexity in asthma. Allergologia et Immunopathologia, 49(2), 191–207. 33641309 10.15586/aei.v49i2.50

[fsn33869-bib-0068] Raoofi, A. , Rezaie, M. J. , Delbari, A. , Ghoreishi, S. A.‐H. , Sichani, P. H. , Maleki, S. , Nasiry, D. , Akhlaghi, M. , Ebrahimi, V. , & Khaneghah, A. M. (2022). Therapeutic potentials of the caffeine in polycystic ovary syndrome in a rat model: Via modulation of proinflammatory cytokines and antioxidant activity. Allergologia et Immunopathologia, 50(6), 137–146. 10.15586/aei.v50i6.715 36335457

[fsn33869-bib-0069] Rasul, A. , Riaz, A. , Wei, W. , Sarfraz, I. , Hassan, M. , Li, J. , Asif, F. , Adem, Ş. , Bukhari, S. A. , Asrar, M. , & Li, X. (2021). *Mangifera indica* extracts as novel PKM2 inhibitors for treatment of triple negative breast cancer. BioMed Research International, 2021, 5514669. 34136566 10.1155/2021/5514669 PMC8175167

[fsn33869-bib-0070] Rawla, P. (2019). Epidemiology of prostate cancer. World Journal of Oncology, 10(2), 63–89. 31068988 10.14740/wjon1191 PMC6497009

[fsn33869-bib-0017] René, D. H. , Ivones, H. B. , Idania, R. G. , Julio, C. R. G. , Olivier, D. W. , Emilie, L. , Ken, D. , Claudina, P. N. , & Wim, B. V. (2020). Anti‐angiogenic effects of mangiferin and mechanism of action in metastatic melanoma. Melanoma Research, 30(1), 39–51. 31651714 10.1097/CMR.0000000000000647

[fsn33869-bib-0071] Rodriguez‐Gonzalez, J. C. , Hernández‐Balmaseda, I. , Declerck, K. , Pérez‐Novo, C. , Logie, E. , Theys, C. , Jakubek, P. , Quiñones‐Maza, O. L. , Dantas‐Cassali, G. , Carlos dos Reis, D. , Camp, G. V. , Paz, M. T. L. , Rodeiro‐Guerra, I. , Delgado‐Hernández, R. , & Vanden Berghe, W. (2021). Antiproliferative, antiangiogenic, and antimetastatic therapy response by mangiferin in a syngeneic immunocompetent colorectal cancer mouse model involves changes in mitochondrial energy metabolism. Frontiers in Pharmacology, 12, 670167. 34924998 10.3389/fphar.2021.670167 PMC8678272

[fsn33869-bib-0107] Rehman, T. U. , El‐Mansi, A. A. , Sadeq, K. A. , Laila, A. A. , Zohaib, S. , Muhammad, A. , Muhammad, R. , Ahmad, B. Z. , & Arfan, Z. M. (2023). Antiparasitic activity of methanolic and ethyl acetate extracts of *Azadirachta indica* against *Haemonchus contortus* . Pakistan Veterinary Journal, 43(1), 199–203.

[fsn33869-bib-0072] Saeed, R. A. , Maqsood, M. , Saeed, R. A. , Muzammil, H. S. , Khan, M. I. , Asghar, L. , Nisa, S. U. , Rabail, R. , & Aadil, R. M. (2022). Plant‐based foods and hepatocellular carcinoma: A review on mechanistic understanding. Critical Reviews in Food Science and Nutrition, 1–34. 10.1080/10408398.2022.2095974 35796706

[fsn33869-bib-0073] Sakthivel, V. , Adeeb, I. Z. , & Vijayabalan, D. (2023). Recent improvements in adult Wilms tumor diagnosis and management: Review of literature. Journal of Kidney Cancer and VHL, 10(3), 32–36. 37583880 10.15586/jkcvhl.v10i3.281 PMC10423726

[fsn33869-bib-0074] Samota, M. K. , Kaur, M. , Sharma, M. , Krishnan, V. , Thakur, J. , Rawat, M. , Phogat, B. , & Guru, P. (2023). Hesperidin from citrus peel waste: Extraction and its health implications. Quality Assurance and Safety of Crops & Foods, 15(2), 71–99.

[fsn33869-bib-0075] Sekhoacha, M. , Riet, K. , Motloung, P. , Gumenku, L. , Adegoke, A. , & Mashele, S. (2022). Prostate cancer review: Genetics, diagnosis, treatment options, and alternative approaches. Molecules, 27(17), 5730. 36080493 10.3390/molecules27175730 PMC9457814

[fsn33869-bib-0076] Shang, H. S. , Chen, C. J. , Shih, Y. L. , Peng, S. F. , Chen, Y. L. , Liu, K. C. , Huang, H. C. , Hsueh, S. C. , Chen, K. W. , Lu, H. F. , Lee, M. F. , Lee, M. Z. , & Lu, K. W. (2021). Mangiferin induces immune responses and evaluates the survival rate in WEHI‐3 cell generated mouse leukemia in vivo. Environmental Toxicology, 36(1), 77–85. 32889744 10.1002/tox.23013

[fsn33869-bib-0077] Simin, J. , Tamimi, R. M. , Callens, S. , Engstrand, L. , & Brusselaers, N. (2020). Menopausal hormone therapy treatment options and ovarian cancer risk: A Swedish prospective population‐based matched‐cohort study. International Journal of Cancer, 147(1), 33–44. 31584190 10.1002/ijc.32706

[fsn33869-bib-0078] Siripongvutikorn, S. , Kluabpet, C. , Anantapan, C. , & Seechamnanturakit, V. (2023). Some factors affecting the bioactive substances of lactogenic tea. Italian Journal of Food Science, 35(3), 75–89.

[fsn33869-bib-0079] Song, W. , Lv, W. , Bi, N. , & Wang, G. (2023). Tectorigenin suppresses the viability of gastric cancer cells in vivo and in vitro. Quality Assurance and Safety of Crops & Foods, 15(3), 117–125.

[fsn33869-bib-0080] Sudduth, C. L. , Castagno, A. , & Maggs, P. (2023). Delayed cardiac metastasis from renal cell carcinoma caused by VHL mutation. Journal of Kidney Cancer and VHL, 10(1), 15–18. 36816596 10.15586/jkcvhl.v10i1.258 PMC9922489

[fsn33869-bib-0111] Swantara, M. D. , Rita, W. S. , Dira, M. A. , & Agustina, K. K. (2022). Effect of the methanol extract of *Annona squamosa* Linn leaf on cervical cancer. International Journal of Veterinary Science, 12(3), 295–301.

[fsn33869-bib-0081] Tan, H.‐Y. , Wang, N. , Li, S. , Hong, M. , Guo, W. , Man, K. , Man, K. , Cheng, C. S. , Chen, Z. , & Feng, Y. (2018). Repression of WT1‐mediated LEF1 transcription by mangiferin governs β‐catenin‐independent Wnt signalling inactivation in hepatocellular carcinoma. Cellular Physiology and Biochemistry, 47(5), 1819–1834. 29953980 10.1159/000491063

[fsn33869-bib-0082] Telange, D. R. , Sohail, N. K. , Hemke, A. T. , Kharkar, P. S. , & Pethe, A. M. (2021). Phospholipid complex‐loaded self‐assembled phytosomal soft nanoparticles: Evidence of enhanced solubility, dissolution rate, ex vivo permeability, oral bioavailability, and antioxidant potential of mangiferin. Drug Delivery and Translational Research, 11(3), 1056–1083. 32696222 10.1007/s13346-020-00822-4

[fsn33869-bib-0083] Tempero, M. A. , Reni, M. , & Riess, H. (2019). APACT: Phase III, multicenter, international, open‐label, randomized trial of adjuvant nab‐paclitaxel plus gemcitabine vs gemcitabine for surgically resected pancreatic adenocarcinoma. Journal of Clinical Oncology, 37(15 Suppl), 4000.

[fsn33869-bib-0084] Thitikornpong, W. , Palanuvej, C. , & Ruangrungsi, N. (2019). In vitro antidiabetic, antioxidation and cytotoxicity activities of ethanolic extract of *Aquilaria crassna* leaves and its active compound; mangiferin. Indian Journal of Traditional Knowledge, 18, 144–150.

[fsn33869-bib-0085] Ullah, M. F. , Ahmad, A. , Bhat, S. H. , Abuduhier, F. M. , Mustafa, S. K. , & Al‐Qirim, T. (2022). Functional profiling of *Achillea fragrantissima* (a perennial edible herb) against human cancer cells and potential nutraceutical impact in neutralizing cell proliferation by interfering with VEGF and NF‐κB signaling pathways. Italian Journal of Food Science, 34(3), 35–47.

[fsn33869-bib-0086] Wang, C. , & Hao, W. (2023). Cardiac arrhythmia and immune response in COVID‐19 patients. Allergologia et Immunopathologia, 51(4), 63–70. 10.15586/aei.v51i4.883 37422781

[fsn33869-bib-0087] Wang, X. , Gu, Y. , Ren, T. , Tian, B. , Zhang, Y. , Meng, L. , & Tang, X. (2013). Increased absorption of mangiferin in the gastrointestinal tract and its mechanism of action by absorption enhancers in rats. Drug Development and Industrial Pharmacy, 39(9), 1408–1413. 22816369 10.3109/03639045.2012.704043

[fsn33869-bib-0088] Wang, X. , Yuwen, T. , & Yanqin, T. (2021). Mangiferin inhibits inflammation and cell proliferation, and activates proapoptotic events via NF‐κB inhibition in DMBA‐induced mammary carcinogenesis in rats. Journal of Environmental Pathology, Toxicology and Oncology, 40(2), 1–9. 10.1615/JEnvironPatholToxicolOncol.2021036057 33822512

[fsn33869-bib-0089] Wani, S. A. , Naik, H. , Wagay, J. A. , Ganie, N. A. , Mulla, M. Z. , & Dar, B. (2022). Mentha: A review on its bioactive compounds and potential health benefits. Quality Assurance and Safety of Crops & Foods, 14(4), 154–168.

[fsn33869-bib-0090] Webb, P. M. , Green, A. C. , & Jordan, S. J. (2017). Trends in hormone use and ovarian cancer incidence in US white and Australian women: Implications for the future. Cancer Causes & Control, 28(5), 365–370. 28233113 10.1007/s10552-017-0868-0

[fsn33869-bib-0091] Wen, W. , Zhu, S. , Ma, R. , Wang, L. , Shen, X. , Li, Y. , Li, Y. , Feng, N. , Wang, L. , Liu, M. , Xie, L. , & Zhang, X. (2022). Correlation analysis of TGF‐β1, MMP‐9, TIMP‐1, IL‐1, IL‐4, IL‐6, IL‐17, and TNF‐α in refractory chronic rhinosinusitis: A retrospective study. Allergologia et Immunopathologia, 50(4), 137–142. 10.15586/aei.v50i4.527 35789413

[fsn33869-bib-0092] Xiang, Y. , Guo, Z. , Zhu, P. , Chen, J. , & Huang, Y. (2019). Traditional Chinese medicine as a cancer treatment: Modern perspectives of ancient but advanced science. Cancer Medicine, 8(5), 1958–1975. 30945475 10.1002/cam4.2108 PMC6536969

[fsn33869-bib-0093] Xiao, W. , Hou, J. , Ma, J. , Yu, B. , Ren, J. , Jin, W. , Wu, J. , Zheng, D. , & Fan, K. (2021). Mangiferin loaded magnetic PCEC microspheres: Preparation, characterization and antitumor activity studies in vitro. Archives of Pharmacal Research, 44(8), 1–7. 10.1007/s12272-014-0485-3 25266232

[fsn33869-bib-0094] Yadav, A. , Kumar, N. , Upadhyay, A. , Singh, A. , Anurag, R. K. , & Pandiselvam, R. (2022). Effect of mango kernel seed starch‐based active edible coating functionalized with lemongrass essential oil on the shelf‐life of guava fruit. Quality Assurance and Safety of Crops & Foods, 14(3), 103–115. 10.15586/qas.v14i3.1094

[fsn33869-bib-0095] Yang, G. , Shang, X. , Cui, G. , Zhao, L. , Zhao, H. , & Wang, N. (2019). Mangiferin attenuated diethynitrosamine‐induced hepatocellular carcinoma in Sprague‐Dawley rats via alteration of oxidative stress and apoptotic pathway. Journal of Environmental Pathology, Toxicology and Oncology, 38(1), 1–12. 10.1615/JEnvironPatholToxicolOncol.2018027392 30806285

[fsn33869-bib-0096] Yang, Y. , Huang, J. , Yan, H. , Li, X. , Zang, P. , Zhang, X. , Xiao, Z. , & Lu, X. (2023). Decreased NK cells in cases of severe adenovirus pneumonia with liver dysfunction in pediatric intensive care unit: Evidence from 330 patients. Allergologia et Immunopathologia, 51(3), 42–48. 37169559 10.15586/aei.v51i3.787

[fsn33869-bib-0097] Yu, L. , Chen, M. , Zhang, R. , & Jin, Z. (2019). Inhibition of cancer cell growth in gemcitabine‐resistant pancreatic carcinoma by mangiferin involves induction of autophagy, endogenous ROS production, cell cycle disruption, mitochondrial mediated apoptosis and suppression of cancer cell migration and invasion. Journal of BUON, 24(4), 1581–1586. 31646812

[fsn33869-bib-0098] Zeng, Z. , Lin, C. , Wang, S. , Wang, P. , Xu, W. , Ma, W. , Wang, J. , Xiang, Q. , Liu, Y. , Yang, J. , Ye, F. , Xie, K. , Xu, J. , Luo, Y. , Liu, S. L. , & Liu, H. (2020). Suppressive activities of mangiferin on human epithelial ovarian cancer. Phytomedicine, 76, 153267. 32570111 10.1016/j.phymed.2020.153267

[fsn33869-bib-0099] Zhang, L. , & Wang, M. (2018). Growth inhibitory effect of mangiferin on thyroid cancer cell line TPC1. Biotechnology and Bioprocess Engineering, 23(6), 649–654.

[fsn33869-bib-0104] Zhang, Y. (2023). Molecular mechanism network pharmacology and bioinformatics research of qingrejiedu decoction in treatment of liver and gallbladder neoplasms. Pakistan Veterinary Journal, 43(3), 477–852.

[fsn33869-bib-0100] Zhu, J. , & Dong, X. (2023). Decursin alleviates LPS‐induced lung epithelial cell injury by inhibiting NF‐κB pathway activation. Allergologia et Immunopathologia, 51(1), 37–43. 10.15586/aei.v51i1.689 36617820

